# The protein-phosphatome of the human malaria parasite *Plasmodium falciparum*

**DOI:** 10.1186/1471-2164-9-412

**Published:** 2008-09-15

**Authors:** Jonathan M Wilkes, Christian Doerig

**Affiliations:** 1 Wellcome Centre for Molecular Parasitology, University of Glasgow, 120 University Place, Glasgow, G12 8TA, Scotland, UK; 2 INSERM U609, Wellcome Centre for Molecular Parasitology, University of Glasgow, 120 University Place, Glasgow, G12 8TA, Scotland, UK

## Abstract

**Background:**

Malaria, caused by the parasitic protist *Plasmodium falciparum*, represents a major public health problem in the developing world. The *P. falciparum *genome has been sequenced, which provides new opportunities for the identification of novel drug targets. We report an exhaustive analysis of the *P. falciparum *genomic database (PlasmoDB) aimed at identifying and classifying all protein phosphatases (PP) in this organism.

**Results:**

Using a variety of bioinformatics tools, we identified 27 malarial putative PP sequences within the four major established PP families, plus 7 sequences that we predict to dephosphorylate "non-protein" substrates. We constructed phylogenetic trees to position these sequences relative to PPs from other organisms representing all major eukaryotic phyla except Cercozoans (for which no full genome sequence is available). Predominant observations were: (i) *P. falciparum *possessed the smallest phosphatome of any of the organisms investigated in this study; (ii) no malarial PP clustered with the tyrosine-specific subfamily of the PTP group (iii) a cluster of 7 closely related members of the PPM/PP2C family is present, and (iv) some *P. falciparum *protein phosphatases are present in clades lacking any human homologue.

**Conclusion:**

The considerable phylogenetic distance between Apicomplexa and other Eukaryotes is reflected by profound divergences between the phosphatome of malaria parasites and those of representative organisms from all major eukaryotic phyla, which might be exploited in the context of efforts for the discovery of novel targets for antimalarial chemotherapy.

## Background

### Eukaryotic protein phosphatases

The reversible phosphorylation of proteins represents a ubiquitous regulatory mechanism for diverse pathways and systems in eukaryotic cells. The process is controlled by a balance between the antagonistic activities of protein kinases, which catalyse the phosphorylation of serine, threonine or tyrosine residues predominantly (reviewed in [1,2]), and more marginally of other residues, notably histidine [3,4], and those of protein phosphatases, which cleave the monophosphate esters from the phosphorylated form of the same residues (reviewed in [4-6]). A large range of kinases have been identified, which seem to have arisen by multiple gene duplication events with subsequent selection [7]. In contrast the number of different protein phosphatase catalytic subunits is much lower than that of kinases, and phosphatases are in general less discriminating than most kinases in substrate selectivity. This lack of specificity combined with high catalytic efficiency suggest that a 'naked' protein phosphatase activity is potentially toxic [6]. The specificity and regulation of many of these enzymes is in fact mediated by accessory proteins (the phosphatase regulatory subunits), a wide variety of which interact with the relatively small repertoire of catalytic subunits (this is not the case for the PTP group, see below). As a consequence, it is speculated that the total number of protein phosphatase holoenzymes involved in regulatory pathways matches, or even exceeds the protein kinase repertoire [8-10]. There are four broad families of protein phosphatases with distinct evolutionary histories:

**1. The PPP group**. PPP sequences (Phospho-Protein Phosphatases) are highly conserved, and constitute perhaps the most highly conserved set of sequences across the eukaryotic kingdom [11,12]. They encode a wide variety of phosphatase activities directed not only at phosphoproteins but at other substrates as well. The dependency of these enzymes on Mn^2+^, Ca^2+ ^and/or Co^2+ ^led to members of this group being called metallophosphatases. The PPP group, which constitutes a subgroup of metallophosphatases, is the most extensively studied type of protein phosphatase. Classically these enzymes were classified into three major groups, PP1, PP2A and PP2B, defined in terms of substrate specificity and inhibitor sensitivity [13]. This classification has been extended in recent years with the identification of a range of sequences related to, but distinct from, PP2A, and a of series of sequences which diverged from the other PPPs early in the evolutionary history of the eukaryotes [6,14]. Thus the PPP family (reviewed in [8]) now comprises as many as eight distinct subtypes of serine/threonine phosphatases: PP1, PP2A, PP2B (calcineurin, PP3), PP4, PP5, PP6, PP7 and the plant-specific BSU subfamily, which is closely related to PP1 and characterised by the presence of a diagnostic Kelch motif [15]. Among these subtypes, PP2, -4 and -6 are closely related to each other and have been grouped in a distinct subfamily [16].

Furthermore, a family of bacterial-like PPP sequences found in eukaryotes (including in *P. falciparum*) has recently been described [17]. Whereas three highly conserved motifs (GDXHG, GDXXDRG and GNH [E/D]) mediating metal coordination in the active centre are considered as the signature of the PPP family, sequences showing no similarities to the known PPP phosphatases beyond the presence of the GDXHG and GDXXDRG motifs were identified in Plants, *Plasmodium*, *Trypanosoma *and some fungi. This revealed the existence in eukaryotes of "non-conventional" branches of the PPP family (reviewed in [17]).

**2. The PPM/PP2C group **comprises a highly diverse, evolutionarily recent set of enzymes with Mg^2+ ^or Mn^2+^-dependent serine/threonine phosphatase activities. The active forms appear to be highly diverse monomeric polypeptides which in many cases possess regulatory domains in C- or N-terminal extensions. A number of defined motifs and conserved residues relate to binding of activating metal ions, water and phosphate groups, as in the PPP type enzymes, but there is no discernable sequence homology between the two groups, despite remarkable structural similarity [18]. A major part of the functions of PP2C (PPM) activities in a variety of species appears to be to modulate stress responses [5,19]. PPM enzymes form part of a superfamily that includes bacterial forms (SpoIIE) and a mitochondrial pyruvate dehydrogenase phosphatase. The PPM family of protein phosphatases is greatly expanded in plants [19].

**3. The PTP (Protein Tyrosine Phosphatase) superfamily**, which is subdivided into three main families: the tyrosine-specific phosphatases, the dual-specificity PTPs (which include the cdc25-like, the Ccdc14 and the MAPK phosphatase groups [20-22]), and the low molecular weight phosphatases [23]. The tyrosine and dual-specificity phosphatases are involved in signalling, cell growth and differentiation, and in the control of cell cycle progression (for example, cdc25 is a major regulator of cyclin-dependent kinase activity [20], and cdc14 regulates mitosis exit by dephosphorylating CDK targets [22]). The enzymes share a common catalytic mechanism mediated by cysteine, arginine and aspartic acid residues. Supplementary domains assist in targeting and substrate specificity [24], in contrast to most other types of phosphatases, which require interaction with regulatory proteins for proper substrate binding.

**4. The NIF group **(**N**LI **i**nteracting **f**actor-like phosphatase) includes the FCP1 (T**F**IIF-associating **C**-terminal domain (CTD) **p**hosphatase 1) and SCP (**S**mall **C**TD **p**hosphatases) [25,26]. These phosphatases are responsible for dephosphorylation of the carboxy-terminal domain (CTD) of RNA polymerase II and interact with the transcription factor TFIIF [27,28]. The function appears to be the dephosphorylation of serine residues within the conserved heptad repeat in the C-terminal, which is required to reactivate the polymerase after termination of transcription. The NIF phosphatases have a DxDx(T/V) motif in the active site [29].

### The case for a Plasmodium phosphatome study

Malaria remains a major public health problem in tropical and subtropical regions, bearing a huge socio-economic impact on affected countries, most of which are in the developing world. Malaria parasites have a complex life cycle. Infection of human beings by *Plasmodium falciparum*, the species responsible for the lethal form of human malaria, begins with the bite of an infected *Anopheles *mosquito, which delivers sporozoites into the bloodstream. These cells establish an infection inside hepatocytes, where they undergo intense multiplication generating several thousand merozoites, a process called exo-erythrocytic schizogony. The merozoites invade erythrocytes, where they also undergo schizogony, the process that is responsible for malaria pathogenesis. Some merozoites, however, arrest the cell cycle and differentiate into male or female gametocytes, which are infective to the mosquito. Once ingested by the insect, the gametocytes develop into gametes and fuse into a zygote. Further development in the mosquito involves a process of sporogony, producing sporozoites that accumulate in the salivary glands and are now ready to infect a new human host (see  for information on malaria).

The study of signalling processes (in particular those involving protein phosphorylation/dephosphorylation) in malaria parasites presents considerable interest, both in terms of fundamental biology (how does a eukaryote that is phylogenetically very distant from model organisms regulate growth, proliferation, differentiation and transition between its complex developmental stages?) and in terms of the search for urgently needed novel drug targets [30,31]. The sequencing of the *P. falciparum *genome [32] and the availability of an interactive genomic database (PlasmoDB, ) [33] have dramatically facilitated the identification of potential targets. Probabilistic models of peptide domains sharing an evolutionary history (Hidden Markov Models, HMMs) permit the rapid scanning of a set of conceptual translations from any organism whose genomic sequence is available [34]. The plasmodial kinome has thus been characterised and highlighted profound divergences from the kinomes of other eukaryotes [35,36]. Although a number of studies have been published on individual phosphatases of malaria parasites (see below), a full "phosphatome" analysis has not been reported, while studies of both the kinomes [37] and phosphatomes [38] of other major parasitic unicellular eukaryotes, the trypanosomatids, have recently been published. Here, we use the Pfam collection of HMMs [39] to investigate the phosphatome of *P. falciparum *in relation to that of members from all major groups of the eukaryotic kingdom [40].

## Results and discussion

### Protein phosphatase-encoding genes in representative organisms from major eukaryotic groups

**Figure 1:**
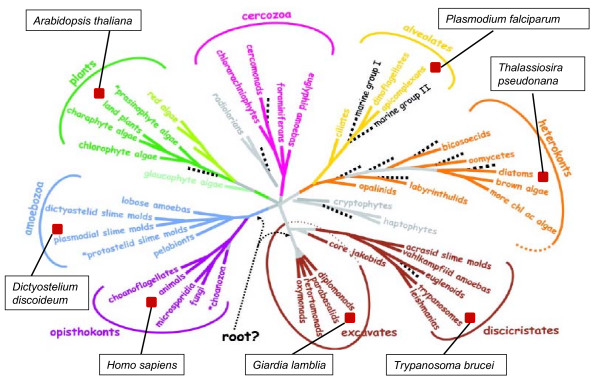
Phylogenetic tree demonstrating the putative relationships between the major types of eukaryotic organisms. Where possible one genome has been selected from each major branch of the tree for comparative studies of the protein phosphatase sequences present. See text for details. Adapted from [40], with permission from the Publisher (AAAS).

**Figure 2:**
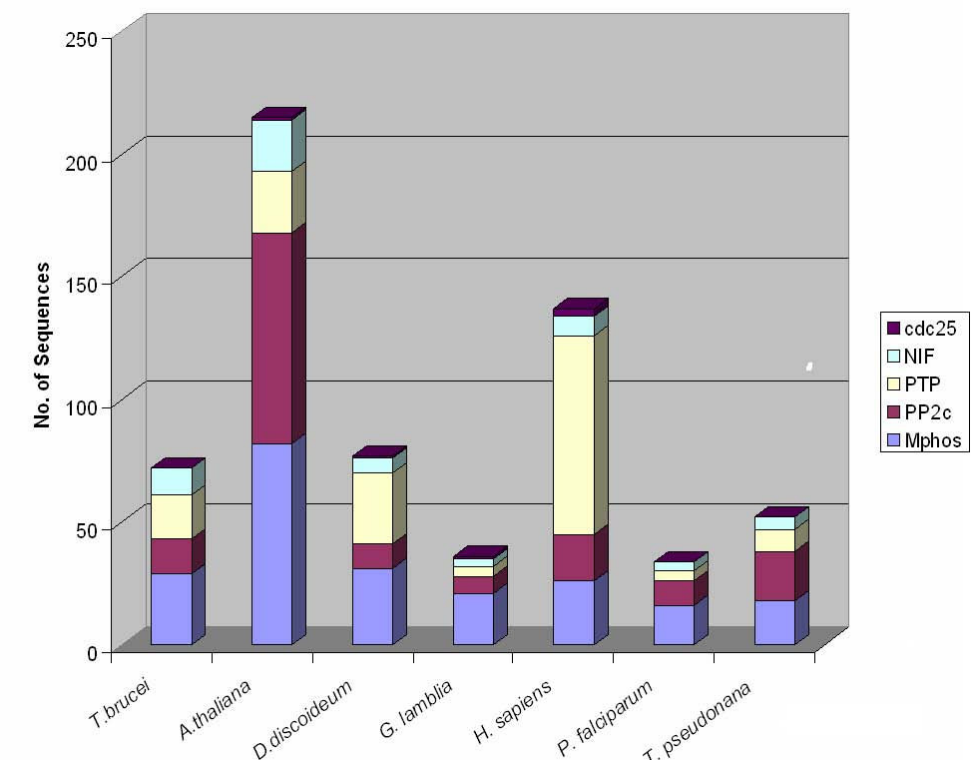
Summary of the genome-wide surveys of the model organisms selected for the comparative studies of protein phosphatase catalytic domains. Results represent HMMER hmmsearch analyses of the conceptual translation set of each genome using the profiles for the four main catalytic domain types. Note the major expansion of PPP (especially PPM type) domains in green plants (*A. thaliana*) and the expansion of PTP type domains in metazoans (*H. sapiens*).

**Figure 3:**
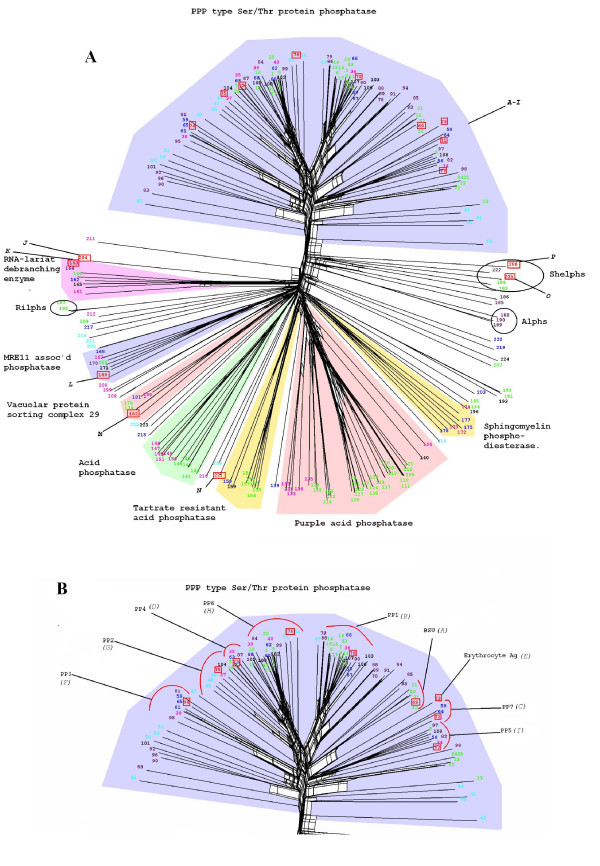
A. Neighbour-Net tree of all Metallophosphatase type (PPP) domains detected in the model genomes. The coloured wedges indicate the distinct clusters defined by the Markov clustering algorithm, which are labelled according to the consensus of annotations available in Swiss-Prot/Trembl/. *P. falciparum *sequences appearing in the tree are labelled by capital letters: A-I *Plasmodium falciparum *PPP type phosphatase domains (see Fig. 3B for identification) J, PF14_0036; K, PF13_0222; L, PF4390w; M, PF14_0064; N, PF14_0614; O, PF14_0660; P, PFL0300c. Sequences J-M are not expected to encode protein phosphatases. The organisms from which the sequences originate are colour-coded as follows: Red and boxed, *P. falciparum *(Alveolates); green, *A. thaliana *(Plants); Blue, *H. Sapiens *(Opisthokonts); Turquoise, *G. lamblia *(Excavates); purple, *T, brucei *(Discicristates); black, *T. pseudonana *(Heterokonts); and magenta, *D. discoideum *(Amoebozoa). B. Enlargement of the region of the Metallophosphatase type domain Neighbour-Net tree of PPP phosphatase domains. Most domain types are labelled according to the human homologue, where present. *P. falciparum *sequences appearing in the tree are labelled by capital letters: A, PF14_0630; B, PF14_0142; C, PF14_0224; D, PFC0595c; E, PF10_0177; F, PF08_0129; G, PFI1245c; H, PFI1360c; I, MAL13P1.274. A high-resolution version of this Figure is available as a PNG file (see Additional file 7). We recommend viewing this file using a graphics programme enabling magnification, such as Microsoft Office Picture Manager.}

HMM profiles defining the diverse phosphatase catalytic domains (see Methods) were used to scan the predicted proteomes of the following organisms, representing all major groups within the eukaryotic kingdom (with the exception or Cercozoans, for which a representative full genome sequence is not available at present): *Homo sapiens *(Opisthokonts), *Dictyostelium discoideum *(Amoebozoa), *Arabidopsis thaliana *(Plants), *P. falciparum *(Alveolates), the diatom *Thalassiosira pseudonana *(Heterokonts), *Trypanosma brucei *(Discicristates) and *Giardia lamblia *(Excavates) (see Fig. 1). This allowed the identification of 633 sequences, with a number of sequences per genome ranging from 34 sequences for *P. falciparum *(the smallest phosphatome in our sample) to 224 sequences for *A. thaliana *(see Additional files 1, 2, 3, 4, 5). The distribution of the various phosphatase families in each phosphatome is illustrated in Fig. 2. The major expansion of all protein phosphatase types, with the exception of PTPs, in *Arabadopsis thaliana *is evident. In *Homo sapiens *the major expansion is in the PTP superfamily. These expansions probably reflect the requirement for flexible and complex intercellular signalling in these multicellular organisms, evidently achieved by distinct evolutionary processes in plants and metazoans. The 34 entries in the *P. falciparum *phosphatome include 7 sequences of the PPP group clustering with subfamilies of phosphatases whose predicted substrates are distinct from phosphoproteins; see Fig. 3). In the next sections we provide a detailed description of *P. falciparum *database mining for each of the 4 major phosphatase groups (PPP, PPM, PTP and NIF).

A high-resolution version of Figure 3 is available as a PNG file (see additional file 7)

### PPP group – Metallophosphatases

#### Constitution of the PPP dataset and construction of a phylogenetic tree

The sixteen catalytic domains conforming to the Pfam profile PF00149 (Metallophosphatase/Calcineurin-like phosphoesterase) identified in *P. falciparum *(Table 1 and Additional file 1), together with those from the other organisms cited above, were subjected to multiple sequence alignment, Markov clustering and Neighbour-Net phylogenetic tree construction (Fig. 3A; the identity and annotation of all sequences displayed in this figure are summarized in Additional file 1). The single largest Markov cluster identified (top of the tree in Fig. 3A, shaded in grey) is clearly separated from the other groupings within the tree. Annotations associated with these sequences indicate that this cluster consists exclusively of serine/threonine phosphatases of the PPP class, whereas the other clusters regroup metallophosphatase classes whose main substrates are not phosphoproteins. Annotations associated with sequences forming distinct clades containing *P. falciparum *entries were used to assign putative function to these enzymes. Of particular interest are two *P. falciparum *sequences (**PF14_0660 **and **PFL0300c**) which cluster close to, but are distinct from, the PPP group. These have been previously identified as similar to bacterial type PPPases (Shelphs) [17].

**Table 1:** A list of the *P. falciparum *sequences in the four groups of protein phosphatases, with the PlasmoDB annotation available at the time of analysis.

**PlasmoDB ID**	**PlasmoDB annotation**	**Motifs**	**Key**	**Refs**
**PPP group**				
				
**PF14_0630**	protein serine/threonine phosphatase		A	42
**PF14_0142**	serine/threonine protein phosphatase, putative		B	45, 46
**PF14_0224**	PP1-like protein serine/threonine phosphatase		C	47, 48
**PFC0595c**	serine/threonine protein phosphatase, putative		D	49
**PF10_0177**	erythrocyte membrane-associated antigen	HT; SP; API	E	
**PF08_0129**	protein phosphatase, putative		F	48
**PFI1245c**	Protein phosphatase-beta		G	52
**PFI1360c**	serine/threonine protein phosphatase, putative		H	
**MAL13P1.274**	serine/threonine protein phosphatase pfPp5		I	55, 56
**PF13_0222**	RNA lariat debranching enzyme, putative		K	
**PFA0390w**	DNA repair exonuclease, putative		L	
**PF14_0064**	vacuolar protein sorting 29, putative		M	
**PF14_0036**	acid phosphatase, putative		J	
**PF14_0660**	hypothetical protein	SP; API	O	
**PFL0300c**	phosphoesterase, putative	SP	P	
**PF14_0614**	hypothetical protein	SP	N	
				
**PPM group**				
				
**MAL13P1.44**	protein phosphatase 2c-like protein, putative		A	
**PFL2365w**	hypothetical protein, conserved		B	
**PF14_0523**	Protein phosphatase 2C, putative		C	
**PFD0505c**	protein phosphatase 2C		D	
**PFE1010w**	protein phosphatase 2c, putative		E	
**PF11_0362**	protein phosphatase, putative		F	
**PF11_0396**	Protein phosphatase 2C		G	59
**MAL8P1.109**	Protein phosphatase 2C, putative		H	
**MAL8P1.108**	protein phosphatase, putative		I	
**PF10_0093**	hypothetical protein		J	
				
**PTP group**				
				
**PF14_0524**	protein phosphatase 7 homolog, putative	API	A	
**PFC0380w**	dual-specificity protein phosphatase, putative		B	67
**PF11_0139**	protein tyrosine phosphatase, putative		C	68
**PF11_0281**	hypothetical protein		D	
				
**NIF group**				
				
**PFE0795c**	nif-like protein, putative		A	
**PF07_0110**	hypothetical protein, conserved		B	
**PF10_0124**	hypothetical protein		C	
**MAL13P1.275**	NLI interacting factor-like phosphatase, putative		D	

Where necessary, an update of the PlasmoDB annotations will be submitted to the database curators. The "Motifs" column reports the targeting signals identified by the following query tools in PlasmoDB: HT, Host Targeting; SP, Signal Peptide; API, Apicoplast targeting. The "Key" column refers to the letters used to identify the sequences in the phylogenetic trees. The last column provides the references for those protein phosphatases that have been described to date in the literature. See text for details.

The "PPP sequences" region of the tree is shown in greater detail in Figure 3B. All families of the PPP type protein phosphatases as identified by Cohen *et al. *[8] are represented in *P. falciparum*, as well as an additional type found only in plants and containing the Kelch motif [41], with which the plasmodial sequence **PF14_0630 **[A] clearly clusters [throughout the article, the capital letter in square brackets following a PlasmoDB identifier refers to the labelling on the figures]; this is the only *P. falciparum *sequence occurring in a PPP group with no homologues in humans.

#### Previously characterised *P. falciparum *metallophosphatases

Many of the plasmodial sequences in this group have been the subjects of previous reports in the literature:

##### PF14_0630 [A] (BSU subfamily)

This protein has been first identified as a PP1-related enzyme, which is confirmed by the position of this sequence in our phylogenetic tree (Fig. 3A); this enzyme was called PfPPαg[42]. Subsequently, it was found that PfPPα has close relatives in plants, which, like the latter enzyme, encode tandem Kelch motifs in their N-terminal extension; the name "PPKLs" (Protein Phosphatases with Kelch-Like domains) was suggested to designate members of this subfamily of PP1 enzymes [15]. The PPKL gene structure is conserved in homologous sequences from the Apicomplexans *Cryptosporidium hominis, Toxoplasma gondii *and *Theileria parva *(one sequence per genome), as well as in the plants *Arabidopsis thaliana *and *Oryza sativa *(3 and 4 occurrences respectively). Kelch motifs form distinctive 'propeller like' tertiary structures proposed to mediate interactions with regulatory subunits [43]. At least one of the three *A. thaliana *gene products is found in the nucleus and appears to be involved in regulating the signal from the brassinosteroid plant hormones [41]. The limited distribution of PPKLs (these proteins have been found only in Plants and Apicomplexa, which is consistent with our phylogenetic tree) is reminiscent with that of other gene families and in line with the proposed photosynthetic ancestry of Apicomplexa [44]. The absence of PPKLs in Opisthokonts suggests PF14_0630 might be a target for parasite-selective inhibition.

##### PF14_0142 [B] (PP1 type)

This protein exhibits the properties of a typical PP1 phospho-serine/threonine phosphatase, and an inhibitor profile consistent with PP1 type activity (IC_50 _values for tautomycin, I-1, I-2 and okadaic acid being 0.8, 400, 7 and 100 nM, respectively) [45]. The protein appears to be expressed in all life cycle stages as judged by Western blot analysis. Microarray analysis indicates a small reduction in expression during the mid-trophozoite stage. RNAi of this sequence resulted in the ablation of PP1 expression, as well as in the impairment of parasite growth (as measured by ^3^H-hypoxanthine incorporation); the subsequent finding that *P. falciparum *does not possess the molecular machinery that mediates RNA interference makes these data difficult to interpret. However, the function of this protein was subsequently confirmed *in vivo *through complementation of a yeast mutant deficient in PP1 activity [46].

##### PF14_0224 [C] (PP7 subgroup)

This protein has been described previously as PfPPJ [47]. The phosphatase activity is okadaic acid-resistant, and catalysis requires Mn^2+ ^but no other cations (Mg^2+ ^or Ca^2+^). Sequence analysis confirmed the presence of the usual metal coordinating, phosphate binding and water activation motifs, but indicated substantial differences from the PP1, PP2A and calcineurin subgroups. This is consistent with our assignment of the sequence to the PP7 subgroup, which appears to have diverged from the other grouping very early in the evolution of the eukaryotes [14]. Similar to PF08_0129 discussed above, subsequent analysis demonstrated the primary PF14_0224 translation product to be much larger than the PP catalytic domain, with two EF-hand motifs that must be occupied by calcium for the enzyme to become fully active [48]. The small size originally predicted for PfPPJ was due to a spurious stop codon in the original cDNA [47], but a fragment corresponding in size to this is apparently produced by post-translational processing detected by Western blotting.

##### PFC0595c [D] (PP2/4/6 type)

This protein has been described previously as PfPPβ [49]. The initial sequence analysis assigned this enzyme to the PP2A group of protein phosphatases. Our analysis suggests, however, that the sequence is a member of the closely related PP2/4/6 family (with closer clustering with the PP4 subgroup), which has been implicated in cell cycle regulation [50]. Although gametocyte-specific expression of PFC0595c mRNA expression was originally reported, microarray data [51] indicate that the gene is expressed at all stages of the asexual cycle, as well as in sporozoites and gametocytes.

##### PF08_0129 [F] (PP3, PP2B or calcineurin subgroup)

Our phylogenetic analysis indicates this sequence to be the only one encoding a calcineurin-type enzyme (can) in the *P. falciparum *genome. A calcineurin type activity (okadaic acid insensitive, calcium dependent) which is non-competitively inhibited by cyclosporine/cyclophilin has been described in the parasite [52] and subsequently attributed to the protein encoded by PF08_0129, which contains a calmodulin-binding domain. The protein appears to be subject to post-translational proteolysis producing a constitutively active core from a large precursor. A putative regulatory subunit of calcineurin (CnB) was identified in the context of the same study [48].

##### PFI1245c [G] (PP2 subgroup)

Previously described by Dobson et al [52], this enzyme activity was potently inhibited by okadaic acid (IC_50 _~ 0.2 nM), and required Mn^2+ ^for activity. These properties led to its classification as a member of the PP2A group. Our phylogenetic analysis supports the assignment of this protein to the PP2 group of protein phosphatases. The same group then identified PfARP (aspartate-rich protein), a plasmodial protein with significant similarity to the I2^PP2A ^family of inhibitors of mammalian PP2A [53]. PfARP was able to inhibit PFI1245c, but none of the four other *P. falciparum *protein phosphatases tested [54].

##### MAL13P1.274 [I] (PP5 subgroup)

This protein has been reported previously independently by two groups [55,56]. The activity is sensitive to nanomolar concentrations of okadaic acid. The sequence of the polypeptide comprises a nuclear targeting sequence at its N-terminus, as well as TPR (tetratricopeptide) repeats, which have an autoinhibitory effect on phosphatase activity; in other systems, this inhibition is relieved by binding unsaturated fatty acids, and indeed, purified recombinant MAL13P1.274 protein, like the native protein enriched from *P. falciparum *extracts, exhibited phosphatase activity that can be enhanced by arachidonic and oleic acids.

#### Uncharacterised *P. falciparum *PPPs

The **PFI1360c **[H] peptide is to our knowledge not described in the literature. Our phylogenetic analysis (Fig 3A) indicates that PFI1360c is most closely related to the PP2/4/6 subgroup, although it emerges at the very base of the cluster, and is therefore relatively divergent from other members of this subgroup. Members of this subgroup are involved in a variety of functions in metazoans, including centrosome maturation, spliceosome assembly, chromatin modification, and regulation of NF-κB and mTOR signalling pathways [16]. As mentioned above, two plasmodial sequences (**PF14_0660 **[O] and **PFL0300c **[P]) cluster close to the "Shewanella-like phosphatases ("Shelphs") group, confirming a prior report that *P. falciparum *possesses two members of this bacterial-like phosphatase family [17]. No functional studies have been reported on these two enzymes; likewise, we are not aware of any published biochemical studies of any of the 7 phosphatases predicted to act on non-protein substrates (sequences J-P in Fig. 3A) present in the tree.

### PPM group

#### Constitution of the PPM dataset

A HMM search of the *P. falciparum *peptide sequence set using the PF00481 (Protein phosphatase 2C) HMM profile produced 10 hits. Markov clustering of the *P. falciparum *sequences, along with the domain-conformant set from the model genomes, was performed to generate a tree.

#### The PPM phylogenetic tree

**Figure 4:**
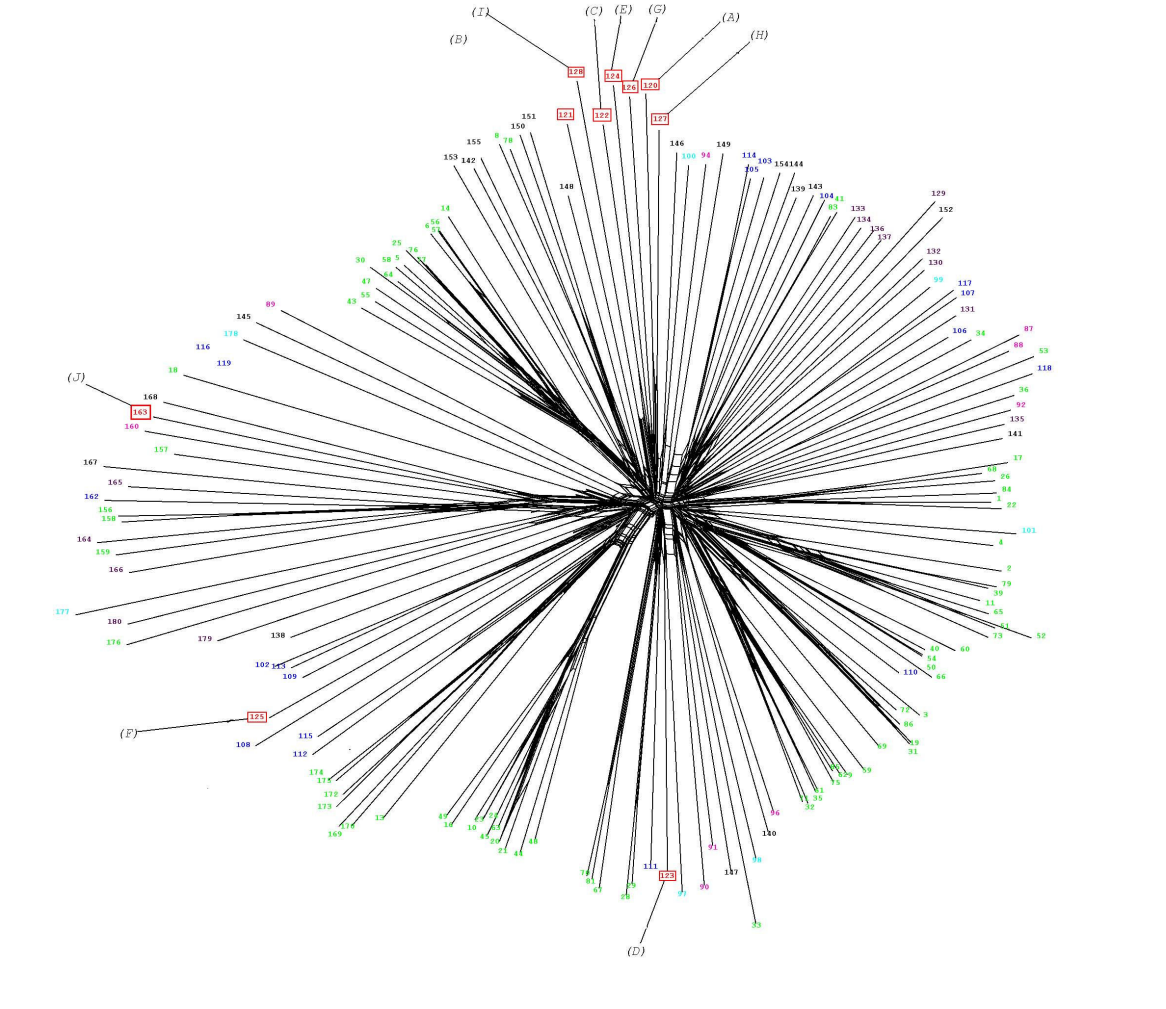
Neighbour-Net tree of all PPM (PP2c) type domains detected in the model genomes. The Markov clustering algorithm defined a single group encompassing the large majority of the sequences; therefore, in contrast to the other figures, no wedges are indicated. *P. falciparum *sequences appearing in the tree are labelled by capital letters: A, MAL13P1.44; B, PFL2365w; C, PF14_0523; D, PFD0505c; E, PFE1010w; F, PF11_0362; G, PF11_0396; H, MAL8P1.109; I, MAL8P1.108; J, PF10_0093. A high-resolution version of this Figure is available as a PNG file (see Additional file 8). We recommend viewing this file using a graphics programme enabling magnification, such as Microsoft Office Picture Manager.

Phylogenetic analysis of the PPM-related sequences was performed as for the PPP group (see above), and the data are summarised in Figure 4, Table 1 and Additional file 2. Annotation of PP2c-conformant sequences is less advanced than that of the PPP family, and a putative function could not be assigned to most sequences. Interestingly, a majority of *P. falciparum *PPM sequences (7/9) cluster together, and these sequences are members of an orthologous group containing only apicomplexan phyla (data not shown), indicating they evolved following early divergence from other Eukaryotes. A sub-grouping of sequences that includes a single *P. falciparum *member (**PF10_0093 **[J]) shows similarity to bacterial SpoIIe domain-containing PP2c-like enzymes involved in the control of sporulation [57]. Other significant groupings consist entirely of *Arabadopsis *sequences, reflecting the very large expansion of this family of phosphatases in plants (see Fig. 2), where PP2c enzymes play major roles in the mediation of stress responses [19,58].

A high-resolution version of this Figure is available as a PNG file (see additional file 8)

#### Previously characterised *P. falciparum *PPMs

The PP2c-type phosphatase **PF11_0396 **[G] is the only PPM from *P. falciparum *reported in the literature [59]. It has been implicated in the regulation of the nucleotide exchange activity of translation elongation factor 1B, antagonising its *in vitr*o phosphorylation by mammalian protein kinase C [60]. In contrast to the monomeric nature of other PPM enzymes, maximal activity is associated with homodimerization of the peptide. The *P. falciparum *PP2c-conformant sequences are found in two regions separated by over 400 residues. Mamoun *et al. *proposed that the peptide contains two distinct PP2c type domains, each capable of enzymatic activity on phosphoserine or – threonine [59]. In this model the dimeric enzyme presents four active sites. Detailed examination of the sequence indicates that only one full set of the conserved functional and structural groups is present in a single polypeptide, and that this complete set is distributed between the two distinct regions. However, evidence that the two peptides interact 'head to tail' may indicate that the regions in the different peptides complement each other, to produce two effective active sites. Such an arrangement may not be uncommon in *Plasmodium*. Two other PPM-related sequences (**PFE1010w **[E] and **MAL8P1.109 **[H]) show evidence of the same split of the PP2c domain, with shorter inserts (200 and 140 residues respectively); there is no experimental data on the function of the latter sequences. It is noteworthy that both are members of the same exclusive Apicomplexan-specific cluster mentioned above that also contains **PF11_0396 **[G].

### PTP Tyrosine phosphatase-like group

#### Constitution of the PTP dataset

Searching the *P. falciparum *peptide set with Pfam-derived HMM profiles of the PTP superfamily identified a small number of sequences conforming to dual-specificity phosphatases (DSPs) [61]: [**PFC0380w, PF14_0524 **(fragment) and **PF14_0525 **(fragment)], and two low scoring hit to tyrosine phosphatases (Y-phosphatases) [62] (**PF11_0139, PF11_0281**) (See Table 1 and Additional file 3). The two fragments PF14_0524 and PF14_0525 are immediately adjacent on the genome, and have similar expression profiles, suggesting that the stop codon separating them may be a misread, or may be read through in translation, as has been shown to be the case for at least one *P. falciparum *gene displaying an internal stop codon [63]. One of the atypical protein kinases of the Apicomplexan-specific FIKK family has the same configuration, with a stop codon interrupting an otherwise complete catalytic domain [35,64]. For further analyses, a hybrid sequence (labelled PF14_052x) was constructed by joining the two sequences. The locus has recently been re-annotated in PlasmoDB: a gene model called "PF14_024_changed" is now proposed, which generates a single predicted polypeptide with a full phosphatase domain encompassing sequences that were previously separated into PF14_0524 and PF14_0525.

#### The PTP phylogenetic tree

**Figure 5:**
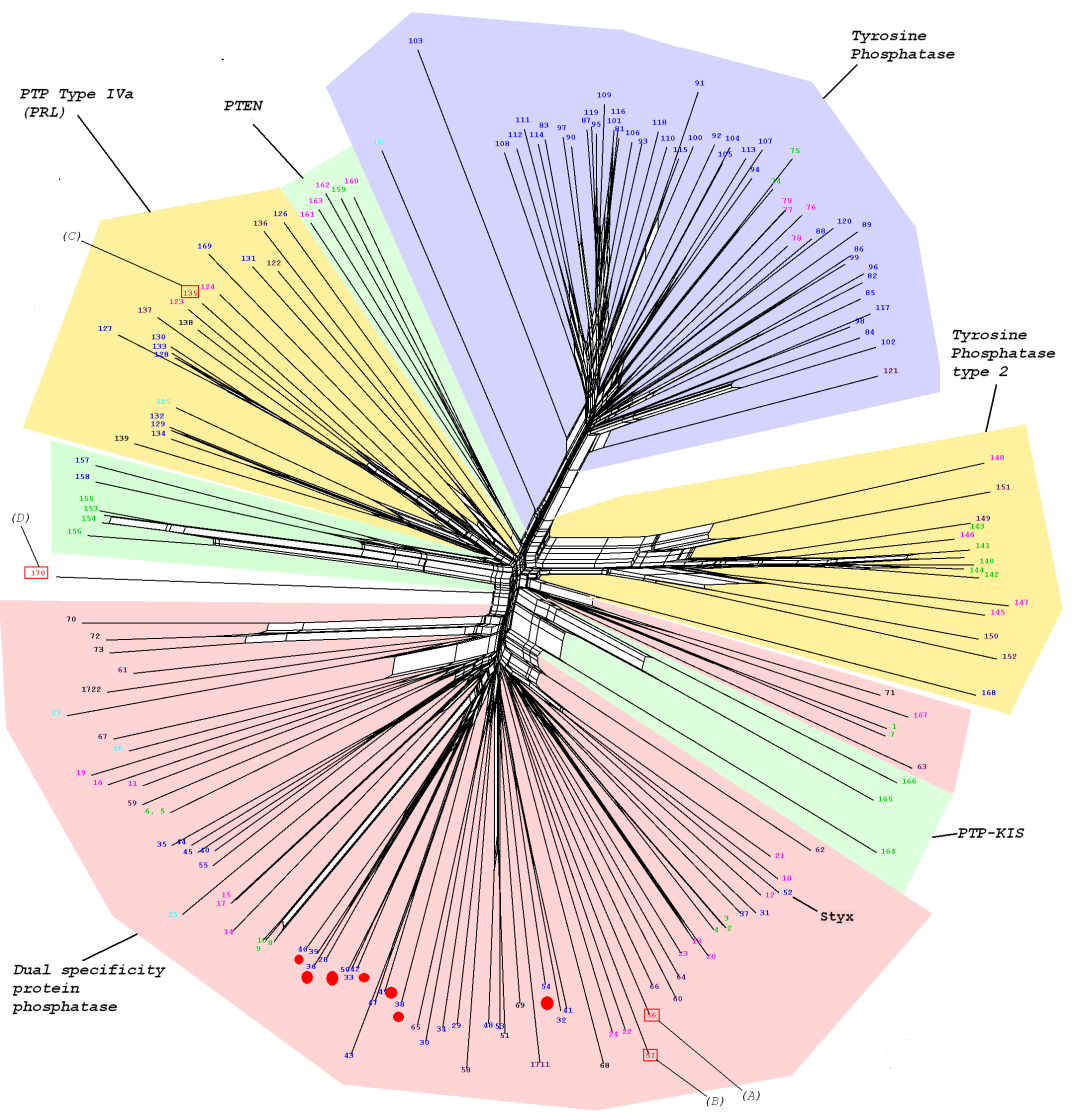
Neighbour-Net tree of all PTP type domains detected in the model genomes. The coloured wedges indicate the distinct clusters defined by the Markov clustering algorithm, which are labelled according to the consensus of annotations available in Swiss-Prot/Trembl. *P. falciparum *sequences appearing in the tree are labelled by capital letters: A, PF14_0525 & PF14_0524; B, PFC0380w; C, PF11_0139; D, PF11_0281. Red dots indicate characterised mammalian MAPK phosphatases. A high-resolution version of this Figure is available as a PNG file (see Additional file 9). We recommend viewing this file using a graphics programme enabling magnification, such as Microsoft Office Picture Manager.

The tree (Fig. 5) confirms that PF14_052x [A] and PFC0380w [B] are clearly DSP-type proteins [61]. DSPs include the enzymes that regulate the activity of the mitogen-activated kinases, which play important role in adaptive responses of eukaryotic cells to extra- or intra-cellular stimuli [61]. The plasmodial kinome encodes two MAPKs, the regulation of which (either positive or negative) is not understood [65]. If phosphatase-mediated negative regulation of MAPKs occurs in the parasite as it does in mammalian cells, PF14_052x and PFC0380w are the most likely candidates in the *Plasmodium *phosphatome to fulfil such a function, in view of their position in the PTP tree; however, this hypothesis remains to be tested experimentally. Interestingly, PF14_052x contains short stretches of positively charged residues near the amino terminus, similar to the "KIM" (Kinase Interaction Motif) found on human MAPK phosphatases and known to mediate binding to the MAPKs [21]. It was shown previously that the activity of one of the plasmodial MAPKs, Pfmap-2, is susceptible to the action of a (mammalian) DSP *in vitro *[66].

A high-resolution version of this Figure is available as a PNG file (see additional file 9)

#### Previously characterised *P. falciparum *PTPs

Two of the four *P. falciparum *PTPs have been the subject of biochemical investigations [67,68]. The PFC0380w [B] polypeptide was assigned to the DSP subgroup, and like other members of this subgroup, contains a functional Zn^2+^-binding domain in addition to its phosphatase catalytic domain. Recombinant PFC0380w exhibits phosphatase activity on both phosphoserine and phosphotyrosine, in line with its assignment to the DSP family.

PF11_0139 [C] belongs to the PRL ("Protein of Regenerating Liver") group [69]. This sequence possesses the CaaX C-terminal motif for farnesylation, a distinguishing feature of this group of phosphatases (the attachment of a farnesyl group generally promotes membrane association to the target protein). It was recently demonstrated that this motif in PF11_0139 (called PfPRL in this study) is indeed the target of farnesyl transferase activity purified from parasite extracts, and that recombinant PfPRL displays phosphatase activity. Interestingly, in merozoites PfPRL co-localises with AMA-1, a membrane-associated protein associated with invasion [68].

To our knowledge, nothing has been published on the other two *P. falciparum *sequences appearing in the tree. It is noteworthy that **PF11_0281 **[D] does not cluster with any branch containing sequences from other Eukaryotes.

Protein tyrosine phosphatase-like proteins (PTPL; Pfam PF04387) constitute a small family of proteins structurally related to PTPs, but the substitution of proline for an essential arginine in the catalytic site renders these polypeptides catalytically inactive. MAL13P1.168 is the only *P. falciparum *sequence containing a PTP-like motif [70]. While the present paper was in revision, a phylogenetic analysis of PTPs in protozoan parasites was published [70], whose conclusion are essentially in agreement with our own data with respect to the representation of *P. falciparum *sequences in the various families of the PTP group.

### The NIF group

**Figure 6:**
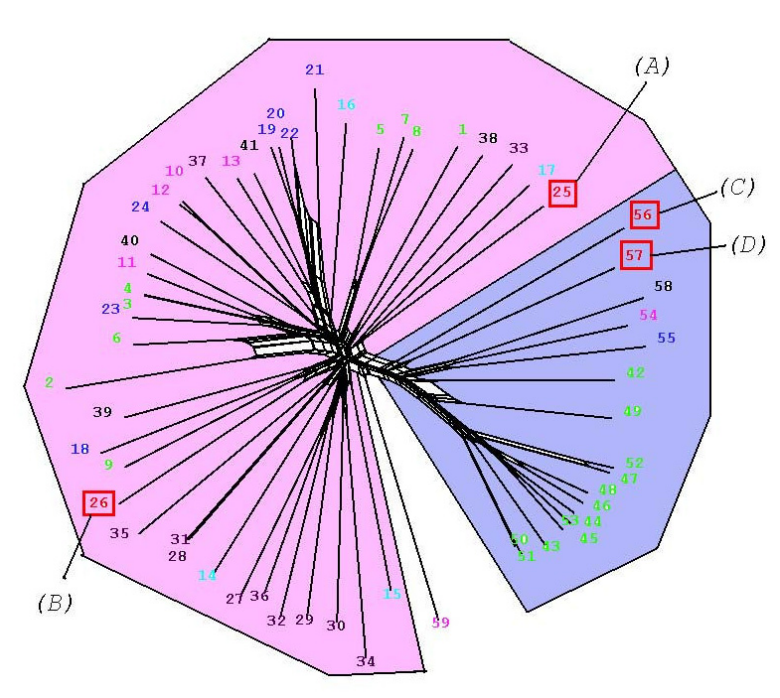
Neighbour-Net tree of all NIF type domains detected in the model genomes. The coloured wedges indicate the distinct clusters defined by the Markov clustering algorithm. *P. falciparum *sequences appearing in the tree are labelled by capital letters: A, PFE0795c; B, PF07_0110; C, PF10_0124; D, MAL13P1.275. A high-resolution version of this Figure is available as a PNG file (see Additional file 10). We recommend viewing this file using a graphics programme enabling magnification, such as Microsoft Office Picture Manager.

Four *P. falciparum *sequences (see Table 1 and Additional file 4) containing domains conforming to the NIF profile were detected. Two of these (**PFE0795c **[A] and **MAL13P1.275 **[D]) have features that are consistent with phosphatase activity (in particular the presence of a putative active site DxDx(T/V) motif), while a third sequence (**PF10_0124 **[C]), although closely related, does not have an intact DxDx(T/V) motif [29], and hence may be catalytically inactive. Both MAL13P1.275 and PF10_0124 possess BRCT domains (the BRCA-C-terminal domain is an evolutionarily conserved phospho-binding domain [71]) diagnostic of Fcp1 phosphatases. PFE0795c is a significantly smaller protein, lacking BRCT domains and hence related to SCP type phosphatases [26,29]. A distinct clade within the phylogenetic tree (Fig. 6) involves NIF type domains associated with the TIM50 sub-unit of the mitochondrial translocase complex. This group includes the *P. falciparum *sequence **PF07_0110 **[B]. It is notable that the DxDx(T/V) motif is disrupted in all these sequences, and thus these proteins are unlikely to possess phosphatase activity.

A high-resolution version of this Figure is available as a PNG file (see additional file 10)

### Missing phosphatase groups

**Figure 7:**
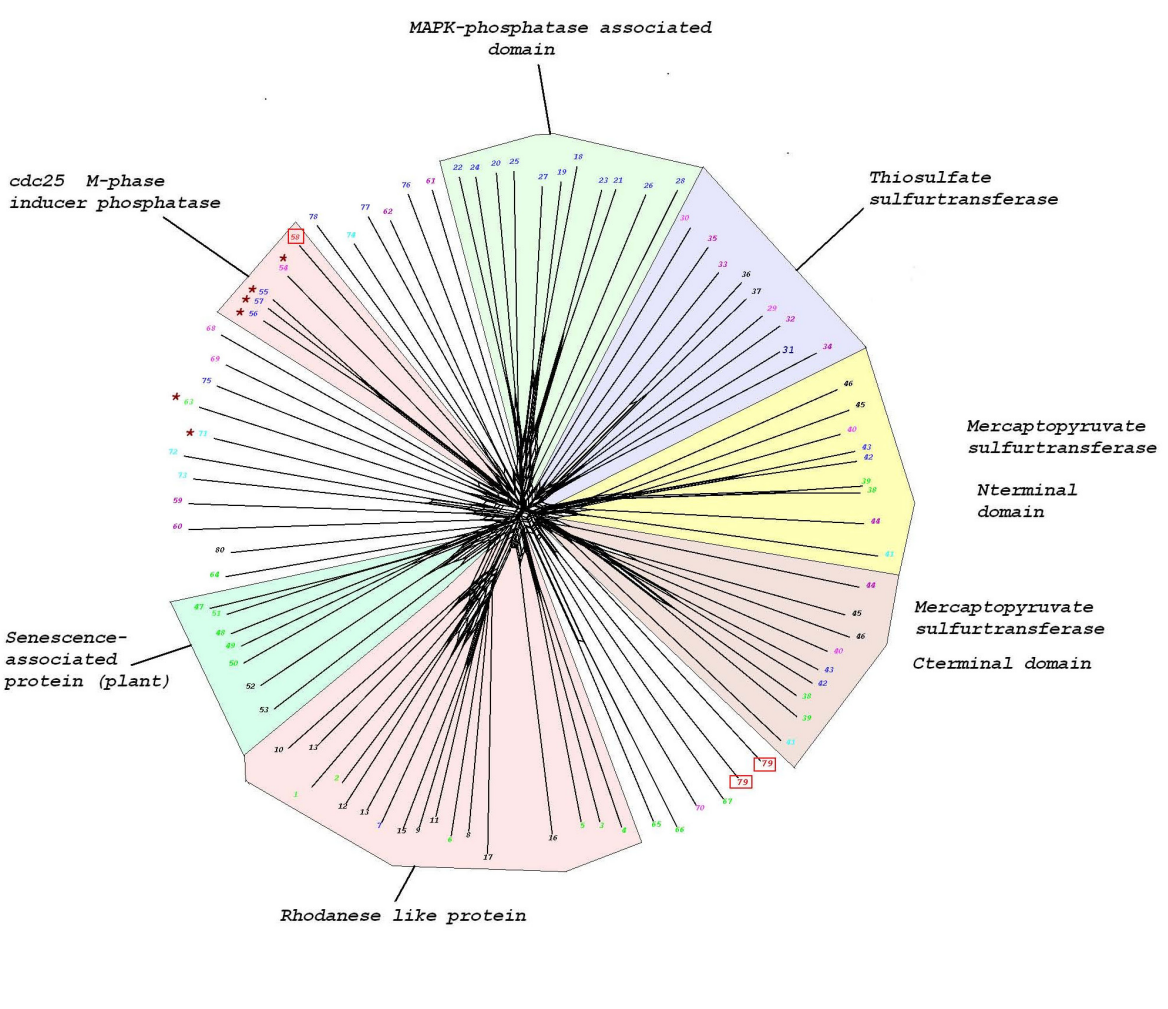
Neighbour-Net tree of all Rhodanese type domains detected in the model genomes. The sequences annotated as "MAPK phosphatase-associated domains" refer to the same polypeptides as the MAPK phosphatases in the PTP tree (Fig. 5), because these proteins contain both a DSP domain and a (non-catalytic) Rhodanese-like domain. The coloured wedges indicate the distinct clusters defined by the Markov clustering algorithm. *P. falciparum *sequences appearing in the tree are shown within red squares. Sequences annotated as cdc25s, containing the CX_5_R catalytic motif, are indicated with an asterisk. See Additional file 6 for an alignment of these sequences with all *P. falciparum *sequences containing a Rhodanese domain. A high-resolution version of this Figure is available as a PNG file (see Additional file 11). We recommend viewing this file using a graphics programme enabling magnification, such as Microsoft Office Picture Manager.

CDC25 enzymes form a distinct group of phosphatases that play a major role in cell cycle control [72]. These enzymes have little sequence similarity to PTPs, except for the presence of the catalytic CX_5_R motif, and appear to have evolved from Rhodanese domains, many of which catalyse sulphur transfer reactions. CDC25s can therefore be identified using a Rhodanese domain HMM profile (PF00581). The cyclin-dependent kinases that mediate cell cycle progression possess conserved threonine and tyrosine residues (T14 and Y15 in human CDK2), whose phosphorylation inactivates the enzyme and causes cell cycle arrest. CDC25 enzymes relieve this block by dephosphorylating these residues. Several *P. falciparum *CDKs display the conserved threonine and tyrosine residues that are the targets of CDC25 in other systems [20,73]. A Rhodanese domain HMM search identified three hits in *P. falciparum*, (Fig. 7), two of which were present on the same polypeptide (**PFL0320w**). The other sequence (**PF13_0027**) clustered with human and *Dictyostelium *CDC25s. We were surprised, however, to notice that this sequence does not contain the CX_5_R motif essential for catalytic activity (see Additional files 5 and 6 for an alignment), and may therefore not encode a functional enzyme. Whether or not plasmodial CDKs are regulated by phosphorylation/dephosphorylation of T14 and Y15 remains to be investigated; either this mechanism of cell cycle control does not operate in malaria parasite (there is to date no evidence that the conserved threonine and tyrosine residues are phosphorylated), or the CDC25 functional homologue is too divergent to be detected.

A high-resolution version of this Figure is available as a PNG file (see additional file 11)

Other protein phosphatase groups for which we found no evidence in *Plasmodium *are the Tyrosine phosphatases (see Fig. 5), the Low Molecular Weight phosphatases [23], the cdc14 phosphatases [22] and the Styx phosphatases. Styx sequences are related to those of PTPs and do recognise phosphotyrosine residues, but are non-catalytic proteins (the catalytic cysteine is replaced by a glycine). Because of their similarity to PTPs, human Styx sequences were picked up in our HMM search and are highlighted on the tree in Fig. 5. However, *P. falciparum *does not possess obvious Styx homologues. Finally, an HMM search of the *P. falciparum *database using the PFAM profile for Myotubularin (MTM) family of lipid phosphatases [74] yielded only hits with very low scores, indicating that the parasite does not encode members of this family.

### Targeting

The putative *P. falciparum *phosphoprotein phosphatase sequences were examined for the presence of signal peptides targeting proteins to various cellular compartments. PlasmoDB records the presence of apicoplast targeting sequences [75], the signal peptides predicted by SignalP [76], and the motif directing proteins to the host erythrocyte [77,78]; the presence of these motifs on PP sequences is indicated in Table 1. In addition the set of sequences was analysed by the PlasMit algorithm  for putative mitochondrial targeting. No sequence demonstrated unequivocal (high stringency) mitochondrial targeting with this algorithm, not even the peptide associated with the TIM50 mitochondrial translocase (PF07_0110); however seven sequences yielded a lower score that is still compatible with mitochondrial targeting (in order of probability: PFE0795c > PF07_0110 > PF11_0362 > MAL8P1.109 > PF10_0093 > MAL13P1.44 > PFI1245c). The (presumably inactive, see above) PF10_0124 is borderline in this respect and is classified as non-mitochondrial. It is relevant to repeat here, as pointed out above, that the PP5-like MAL13P1.174 possesses a nuclear localisation signal. It is important to emphasise (i) that the presence or absence of targeting motifs on any sequence is dependent on gene predictions and can vary with database re-annotations, and (ii) that the functionality of such motifs should be verified experimentally.

### Associated domains and motifs

In addition to the accessory domain instances described earlier, the only sequence containing associated domains with homologues in other organisms is that annotated as erythrocyte membrane-associated antigen (**PF10_0177**). In the sequence we originally downloaded and used in our analyses, this large polypeptide had an EF-hand domain and a putative acid protease domain in addition to the phosphatase domain. In a recent re-annotation of this locus, however, the open reading frames encoding the phosphatase and protease domains are proposed to be split and expressed as distinct genes. Other domain combinations are discussed above.

## Conclusion

A "protein phosphatase" keyword query on PlasmoDB yielded 18 entries in the annotated *P. falciparum *genome, to be compared to the 27 PP sequences we retrieved using HHM searches. Using "phosphatase" as a query, PlasmoDB yielded 30 entries, a very similar number to the total number (34) of sequences of enzymes that phosphorylate proteins and non-protein substrates we found using HMMs. This indicates that in this instance the Plasmodb annotation is remarkably accurate, but detailed annotation of these genes can be improved – we are addressing this issue with the database curators.

We based our approach on HMM searches using established profiles, which would of course miss any "cryptic", non-HMM-conforming enzymes. The list we propose here must therefore be viewed as the minimal complement of functional protein phosphatases.

The ratio of protein kinases to protein phosphatases in *P. falciparum *is close to 2:1, in line with the smaller numbers of phosphatase catalytic domains (compared with those of kinase catalytic domains) present in other eukaryotes. The *A. thaliana *phosphatome contains a large number of PPMs (linked to modulation of stress responses through the MAPK pathway [19,21,54]), which may be linked to the observation that Plant genomes contain a much larger number of genes coding for receptor kinases than other organisms (reviewed in [79]). Similarly, PTPs linked to intercellular signalling, and antagonistic to a large repertoire of tyrosine kinases are vastly expanded in the mammalian phosphatome (Fig. 2). In contrast, the complement of phosphatases in *P. falciparum *does not include any markedly expanded family other than the 7-member cluster of PPMs described above, despite a major expansion of FIKK type kinases observed previously [35,64]. Interestingly, the diversity of PP types represented in the malarial phosphatome is relatively high despite a comparatively small number of enzymes, which is explained by our observation that subtypes in the four PP groups are represented by one member only; this is particularly apparent in the PPP group, where subtypes are frequently represented by one member only. Thus the parasite maintains a large functional capability despite a small phosphatome. We have not addressed here the identification of protein phosphatase regulatory subunits. Undoubtedly the parasite possesses many such polypeptides, which are likely to considerably increase functional diversity (reviewed in [5]). It will be fascinating to explore the functional implications, in terms of both specific biochemical processes (signalling, motility, cell cycle and transcription control, transport, among many others) and overall parasite development, of the antagonism between specific instances of protein phosphorylation and dephosphorylation. Importantly, phosphatases are gaining recognition as potential targets for chemotherapeutic intervention [80], and have been estimated to represent 4% of the druggable human genome; in particular, PTPs appear an important new target for cancer therapy, notably for melanoma (reviewed in [81]). Thus, the *P. falciparum *phosphatases, like the plasmodial protein kinases [31], might well, in the near future, join the cohort of potential targets for novel antimalarials.

## Methods

### Selection of Hidden Markov Models for protein phosphatase catalytic domains

In contrast to the catalytic domains of the protein kinase superfamily, the vast majority of which conform to a single HMM profile (Pfam database entry PF00069; Pkinase), the diversity of protein phosphatases is reflected by the presence of 7 distinct Pfam profiles defining catalytic domains with protein phosphatase activity. Please see table 2

**Table 2:** Profiles used, unaltered, to mine the various genomes for conformant sequences.

PF00102	Y-phosphatase	Tyrosine
PF01451	LMWP	Tyrosine
PF00481	PP2c	Serine/Threonine
PF00782	DSP	Tyrosine/Serine/Threonine
PF03162	Y-phosphatase2	Tyrosine
PF03031	NIF phosphatase	Serine/Threonine
PF00581	Rhodanese domain (CDC25)	Threonine/Tyrosine
PF00149	Metallophosphatase	Serine/Threonine; Many proteins with catalytic sites conforming to the PF00149 profile hydrolyse phosphate esters on substrates other than phosphoproteins.

The above profiles were downloaded from Pfam [39,82], and used unaltered to mine the various genomes for conformant sequences.

Two tyrosine phosphatases (PF00102; Y-phosphatase, PF03162; Y-phosphatase2), and a dual-specificity serine/threonine/tyrosine phosphatase activity (PF00782; DSP) are closely related and are grouped in a single clan, the protein tyrosine-phosphatase superfamily (also referred to as PTP) [82]. An additional low molecular weight tyrosine phosphatase [PF01451] with limited sequence similarity, but possessing the characteristic PTP motif, is also listed. Serine-threonine phosphatase activities are found in two distinct groups: a highly conserved group conforming to the Metallophosphatase family (Pfam profilePF00149; note that this family includes a wide range of phosphatase activities in addition to protein phosphatases; the protein phosphatase activities are classified as PPP type) and a structurally unrelated (though catalytically similar) group, the PP2C family (Pfam profile PF00481, PPM).

### Identification of catalytic domains

Catalytic domains were identified by use of the hmmsearch option of HMMER [34] using Hidden Markov Profiles appropriate to the domain of interest using moderately stringent criteria (Expect value [-E] of 10^-3^, database record number [-Z] 100000). The initial search used the global model for each domain type, although the local model was subsequently used where appropriate if multiple or fragmented domains were found.

### Extraction of profile conformant sequences (PP domain plus short flanking sequences)

Peptide sequences were aligned under the guidance of an appropriate HMM profile [34] using the hmmalign option of HMMER. Alignment output in ClustalW format was trimmed down to those blocks encompassing match states to the profile and ungapped Fasta formatted sequences extracted from this sub-set of the alignment. (T_coffee seq_reformat option).

### Multiple sequence alignment

MSA of a given sequence set was performed by three independent methods; ClustalW [83], t-coffee [84] and hmmalign [34] guided by the appropriate profile. The alignments used the default settings for each method. Alignments were combined under t-coffee, and quality of alignment assessed.

### Clustering of model genome peptide sequences with identified *P. falciparum *sequences

The eukaryotic kingdom is extremely diverse, and molecular analysis confidently identifies eight major groups within this diversity. For a broad phylogenetic and evolutionary analysis of the protein phosphatases present in *P. falciparum *the translated gene products (June 2006 versions) were downloaded from completed genome projects of the following species:

*Homo sapiens *(Opisthokonts)



*Dictyostelium discoideum *(amoebazoa)



*Arabidopsis thaliana *(viridiplantae)



*Plasmodium falciparum *(alveolates)



*Thalassiosira pseudonana *(heterokonts)



*Trypanosoma brucei *(discicristates)



*Giardia lamblia *(excavates)



At the time of writing, no genomic information was available for any member of the cercozoan group. Translated peptide sets for the six model genomes were combined and subjected to the HMMER hmmsearch option using the above criteria. Identified sequences were retrieved using the blast [85] fastacmd option, and domain conformant subsequences extracted. Appropriate subsequences derived from *P. falciparum *sequences were added to the dataset. An all against all blastp (-e 0.01) of the sequence set was performed, and Markov clustering of the output performed under control of the Tribe package [86]. The inflation parameter (-I) was 1.7, a value which demonstrates a reasonable discrimination without fragmenting clusters to an unusable degree

### Phylogenetic analysis

High quality Multiple Sequence Alignments of the catalytic domains were prepared as described above, and columns displaying low consistency (score < 5) or significant numbers of gaps (> 15%) removed. Alternate neighbour joining phylogenies were visualised using Neighbour-Net, implemented on SplitsTree version 4 [87].

## Authors' contributions

JW performed all the database searches for PP-related sequences, constructed the phylogenetic trees, wrote the Method part of the manuscript, performed literature searches; CD supervised the study and the preparation of the manuscript. Both authors read and approved the manuscript.

## Supplementary Material

Additional file 1 List of PPP-conformant sequences. See legend within the file. Click here for file

Additional file 2 List of PPP-conformant sequences. See legend within the file. Click here for file

Additional file 3 List of PTP-conformant sequences. See legend within the file. Click here for file

Additional file 4 List of NIF-conformant sequences. See legend within the file. Click here for file

Additional file 5 List of Rhodanese-conformant sequences. See legend within the file. Click here for file

Additional file 7 High resolution file of Fig. 3. See legend to Fig. 3. Click here for file

Additional file 8 High resolution file of Fig. 4. See legend to Fig. 4. Click here for file

Additional file 9 High resolution file of Fig. 5. See legend to Fig. 5 Click here for file

Additional file 10 High resolution file of Fig. 6. See legend to Fig. 6. Click here for file

Additional file 6 Alignment of Rhodanese-containing domains. See legend within the file. Click here for file

Additional file 11 High resolution file of Fig. 7. See legend to Fig. 7. Click here for file

## References

[B1] Hanks SK (2003). Genomic analysis of the eukaryotic protein kinase superfamily: a perspective. Genome Biol.

[B2] Hanks SK, Quinn AM (1991). Protein kinase catalytic domain sequence database: identification of conserved features of primary structure and classification of family members. Methods Enzymol.

[B3] West AH, Stock AM (2001). Histidine kinases and response regulator proteins in two-component signaling systems. Trends Biochem Sci.

[B4] Klumpp S, Krieglstein J (2005). Reversible phosphorylation of histidine residues in vertebrate proteins. Biochim Biophys Acta.

[B5] Barford D, Das AK, Egloff MP (1998). The structure and mechanism of protein phosphatases: insights into catalysis and regulation. Annu Rev Biophys Biomol Struct.

[B6] Gallego M, Virshup DM (2005). Protein serine/threonine phosphatases: life, death, and sleeping. Curr Opin Cell Biol.

[B7] Miranda-Saavedra D, Barton GJ (2007). Classification and functional annotation of eukaryotic protein kinases. Proteins.

[B8] Cohen PT (1997). Novel protein serine/threonine phosphatases: variety is the spice of life. Trends Biochem Sci.

[B9] Cohen PT, Chen MX, Armstrong CG (1996). Novel protein phosphatases that may participate in cell signaling. Adv Pharmacol.

[B10] Bollen M (2001). Combinatorial control of protein phosphatase-1. Trends Biochem Sci.

[B11] Orgad S, Brewis ND, Alphey L, Axton JM, Dudai Y, Cohen PT (1990). The structure of protein phosphatase 2A is as highly conserved as that of protein phosphatase 1. FEBS Lett.

[B12] Barton GJ, Cohen PT, Barford D (1994). Conservation analysis and structure prediction of the protein serine/threonine phosphatases. Sequence similarity with diadenosine tetraphosphatase from Escherichia coli suggests homology to the protein phosphatases. Eur J Biochem.

[B13] Wera S, Hemmings BA (1995). Serine/threonine protein phosphatases. Biochem J.

[B14] Andreeva AV, Kutuzov MA (2001). PPP family of protein Ser/Thr phosphatases: two distinct branches?. Mol Biol Evol.

[B15] Kutuzov MA, Andreeva AV (2002). Protein Ser/Thr phosphatases with kelch-like repeat domains. Cell Signal.

[B16] Cohen PT, Philp A, Vazquez-Martin C (2005). Protein phosphatase 4 – from obscurity to vital functions. FEBS Lett.

[B17] Andreeva AV, Kutuzov MA (2004). Widespread presence of "bacterial-like" PPP phosphatases in eukaryotes. BMC Evol Biol.

[B18] Das AK, Helps NR, Cohen PT, Barford D (1996). Crystal structure of the protein serine/threonine phosphatase 2C at 2.0 A resolution. Embo J.

[B19] Schweighofer A, Hirt H, Meskiene I (2004). Plant PP2C phosphatases: emerging functions in stress signaling. Trends Plant Sci.

[B20] Boutros R, Dozier C, Ducommun B (2006). The when and wheres of CDC25 phosphatases. Curr Opin Cell Biol.

[B21] Owens DM, Keyse SM (2007). Differential regulation of MAP kinase signalling by dual-specificity protein phosphatases. Oncogene.

[B22] Trinkle-Mulcahy L, Lamond AI (2006). Mitotic phosphatases: no longer silent partners. Curr Opin Cell Biol.

[B23] Raugei G, Ramponi G, Chiarugi P (2002). Low molecular weight protein tyrosine phosphatases: small, but smart. Cell Mol Life Sci.

[B24] Fauman EB, Saper MA (1996). Structure and function of the protein tyrosine phosphatases. Trends Biochem Sci.

[B25] Yeo M, Lin PS (2007). Functional characterization of small CTD phosphatases. Methods Mol Biol.

[B26] Yeo M, Lin PS, Dahmus ME, Gill GN (2003). A novel RNA polymerase II C-terminal domain phosphatase that preferentially dephosphorylates serine 5. J Biol Chem.

[B27] Kobor MS, Greenblatt J (2002). Regulation of transcription elongation by phosphorylation. Biochim Biophys Acta.

[B28] Suh MH, Ye P, Zhang M, Hausmann S, Shuman S, Gnatt AL, Fu J (2005). Fcp1 directly recognizes the C-terminal domain (CTD) and interacts with a site on RNA polymerase II distinct from the CTD. Proc Natl Acad Sci USA.

[B29] Hausmann S, Shuman S (2003). Defining the active site of Schizosaccharomyces pombe C-terminal domain phosphatase Fcp1. J Biol Chem.

[B30] Tilley L, Davis TM, Bray PG (2006). Prospects for the treatment of drug-resistant malaria parasites. Future Microbiol.

[B31] Doerig C, Meijer L (2007). Antimalarial drug discovery: targeting protein kinases. Expert Opin Ther Targets.

[B32] Gardner MJ, Hall N, Fung E, White O, Berriman M, Hyman RW, Carlton JM, Pain A, Nelson KE, Bowman S (2002). Genome sequence of the human malaria parasite Plasmodium falciparum. Nature.

[B33] Stoeckert CJ, Fischer S, Kissinger JC, Heiges M, Aurrecoechea C, Gajria B, Roos DS (2006). PlasmoDB v5: new looks, new genomes. Trends Parasitol.

[B34] Eddy SR (1998). Profile hidden Markov models. Bioinformatics.

[B35] Ward P, Equinet L, Packer J, Doerig C (2004). Protein kinases of the human malaria parasite Plasmodium falciparum: the kinome of a divergent eukaryote. BMC Genomics.

[B36] Anamika, Srinivasan N, Krupa A (2005). A genomic perspective of protein kinases in Plasmodium falciparum. Proteins.

[B37] Parsons M, Worthey EA, Ward PN, Mottram JC (2005). Comparative analysis of the kinomes of three pathogenic trypanosomatids: Leishmania major, Trypanosoma brucei and Trypanosoma cruzi. BMC Genomics.

[B38] Brenchley R, Tariq H, McElhinney H, Szoor B, Huxley-Jones J, Stevens R, Matthews K, Tabernero L (2007). The TriTryp phosphatome: analysis of the protein phosphatase catalytic domains. BMC Genomics.

[B39] Bateman A, Birney E, Cerruti L, Durbin R, Etwiller L, Eddy SR, Griffiths-Jones S, Howe KL, Marshall M, Sonnhammer EL (2002). The Pfam protein families database. Nucleic Acids Res.

[B40] Baldauf SL (2003). The deep roots of eukaryotes. Science.

[B41] Mora-Garcia S, Vert G, Yin Y, Cano-Delgado A, Cheong H, Chory J (2004). Nuclear protein phosphatases with Kelch-repeat domains modulate the response to brassinosteroids in Arabidopsis. Genes Dev.

[B42] Li JL, Baker DA (1998). A putative protein serine/threonine phosphatase from Plasmodium falciparum contains a large N-terminal extension and five unique inserts in the catalytic domain. Mol Biochem Parasitol.

[B43] Adams J, Kelso R, Cooley L (2000). The kelch repeat superfamily of proteins: propellers of cell function. Trends Cell Biol.

[B44] Waller RF, McFadden GI (2005). The apicoplast: a review of the derived plastid of apicomplexan parasites. Curr Issues Mol Biol.

[B45] Kumar R, Adams B, Oldenburg A, Musiyenko A, Barik S (2002). Characterisation and expression of a PP1 serine/threonine protein phosphatase (PfPP1) from the malaria parasite, Plasmodium falciparum: demonstration of its essential role using RNA interference. Malar J.

[B46] Bhattacharyya MK, Hong Z, Kongkasuriyachai D, Kumar N (2002). Plasmodium falciparum protein phosphatase type 1 functionally complements a glc7 mutant in Saccharomyces cerevisiae. Int J Parasitol.

[B47] Dobson S, Bracchi V, Chakrabarti D, Barik S (2001). Characterization of a novel serine/threonine protein phosphatase (PfPPJ) from the malaria parasite, Plasmodium falciparum. Mol Biochem Parasitol.

[B48] Kumar R, Musiyenko A, Oldenburg A, Adams B, Barik S (2004). Post-translational generation of constitutively active cores from larger phosphatases in the malaria parasite, Plasmodium falciparum: implications for proteomics. BMC Mol Biol.

[B49] Li JL, Baker DA (1997). Protein phosphatase beta, a putative type-2A protein phosphatase from the human malaria parasite Plasmodium falciparum. Eur J Biochem.

[B50] Bastians H, Ponstingl H (1996). The novel human protein serine/threonine phosphatase 6 is a functional homologue of budding yeast Sit4p and fission yeast ppe1, which are involved in cell cycle regulation. J Cell Sci.

[B51] Le Roch KG, Zhou Y, Blair PL, Grainger M, Moch JK, Haynes JD, De La Vega P, Holder AA, Batalov S, Carucci DJ (2003). Discovery of gene function by expression profiling of the malaria parasite life cycle. Science.

[B52] Dobson S, May T, Berriman M, Del Vecchio C, Fairlamb AH, Chakrabarti D, Barik S (1999). Characterization of protein Ser/Thr phosphatases of the malaria parasite, Plasmodium falciparum: inhibition of the parasitic calcineurin by cyclophilin-cyclosporin complex. Mol Biochem Parasitol.

[B53] Li M, Guo H, Damuni Z (1995). Purification and characterization of two potent heat-stable protein inhibitors of protein phosphatase 2A from bovine kidney. Biochemistry.

[B54] Dobson S, Kumar R, Bracchi-Ricard V, Freeman S, Al-Murrani SW, Johnson C, Damuni Z, Chakrabarti D, Barik S (2003). Characterization of a unique aspartate-rich protein of the SET/TAF-family in the human malaria parasite, Plasmodium falciparum, which inhibits protein phosphatase 2A. Mol Biochem Parasitol.

[B55] Dobson S, Kar B, Kumar R, Adams B, Barik S (2001). A novel tetratricopeptide repeat (TPR) containing PP5 serine/threonine protein phosphatase in the malaria parasite, Plasmodium falciparum. BMC Microbiol.

[B56] Lindenthal C, Klinkert MQ (2002). Identification and biochemical characterisation of a protein phosphatase 5 homologue from Plasmodium falciparum. Mol Biochem Parasitol.

[B57] Carniol K, Ben-Yehuda S, King N, Losick R (2005). Genetic dissection of the sporulation protein SpoIIE and its role in asymmetric division in Bacillus subtilis. J Bacteriol.

[B58] Chakraborty N, Ohta M, Zhu JK (2007). Recognition of a PP2C interaction motif in several plant protein kinases. Methods Mol Biol.

[B59] Mamoun CB, Sullivan surDJ Jr, Banerjee R, Goldberg DE (1998). Identification and characterization of an unusual double serine/threonine protein phosphatase 2C in the malaria parasite Plasmodium falciparum. J Biol Chem.

[B60] Mamoun CB, Goldberg DE (2001). Plasmodium protein phosphatase 2C dephosphorylates translation elongation factor 1beta and inhibits its PKC-mediated nucleotide exchange activity in vitro. Mol Microbiol.

[B61] Roma-Mateo C, Rios P, Tabernero L, Attwood TK, Pulido R (2007). A novel phosphatase family, structurally related to dual-specificity phosphatases, that displays unique amino acid sequence and substrate specificity. J Mol Biol.

[B62] Dewang PM, Hsu NM, Peng SZ, Li WR (2005). Protein tyrosine phosphatases and their inhibitors. Curr Med Chem.

[B63] Bischoff E, Guillotte M, Mercereau-Puijalon O, Bonnefoy S (2000). A member of the Plasmodium falciparum Pf60 multigene family codes for a nuclear protein expressed by readthrough of an internal stop codon. Mol Microbiol.

[B64] Schneider AG, Mercereau-Puijalon O (2005). A new Apicomplexa-specific protein kinase family: multiple members in Plasmodium falciparum, all with an export signature. BMC Genomics.

[B65] Dorin D, Semblat JP, Poullet P, Alano P, Goldring D, Whittle C, Patterson S, Whittle C, Chakrabarti D, Doerig C (2005). PfPK7, an atypical MEK-related protein kinase, reflects the absence of typical three-component MAP kinase pathways in the human malaria parasite Plasmodium falciparum. Mol Microbiol.

[B66] Dorin D, Alano P, Boccaccio I, Ciceron L, Doerig C, Sulpice R, Parzy D, Doerig C (1999). An atypical mitogen-activated protein kinase (MAPK) homologue expressed in gametocytes of the human malaria parasite Plasmodium falciparum. Identification of a MAPK signature. Journal of Biological Chemistry.

[B67] Kumar R, Musiyenko A, Cioffi E, Oldenburg A, Adams B, Bitko V, Krishna SS, Barik S (2004). A zinc-binding dual-specificity YVH1 phosphatase in the malaria parasite, Plasmodium falciparum, and its interaction with the nuclear protein, pescadillo. Mol Biochem Parasitol.

[B68] Pendyala PR, Ayong L, Eatrides J, Schreiber M, Pham C, Chakrabarti R, Fidock DA, Allen CM, Chakrabarti D (2008). Characterization of a PRL protein tyrosine phosphatase from Plasmodium falciparum. Mol Biochem Parasitol.

[B69] Stephens BJ, Han H, Gokhale V, Von Hoff DD (2005). PRL phosphatases as potential molecular targets in cancer. Mol Cancer Ther.

[B70] Andreeva AV, Kutuzov MA (2008). Protozoan protein tyrosine phosphatases. Int J Parasitol.

[B71] Yu X, Chini CC, He M, Mer G, Chen J (2003). The BRCT domain is a phospho-protein binding domain. Science.

[B72] Rudolph J (2007). Cdc25 phosphatases: structure, specificity, and mechanism. Biochemistry.

[B73] Doerig C, Endicott J, Chakrabarti D (2002). Cyclin-dependent kinase homologues of Plasmodium falciparum. Int J Parasitol.

[B74] Clague MJ, Lorenzo O (2005). The myotubularin family of lipid phosphatases. Traffic.

[B75] Foth BJ, Ralph SA, Tonkin CJ, Struck NS, Fraunholz M, Roos DS, Cowman AF, McFadden GI (2003). Dissecting apicoplast targeting in the malaria parasite Plasmodium falciparum. Science.

[B76] Emanuelsson O, Brunak S, von Heijne G, Nielsen H (2007). Locating proteins in the cell using TargetP, SignalP and related tools. Nat Protoc.

[B77] Marti M, Good RT, Rug M, Knuepfer E, Cowman AF (2004). Targeting malaria virulence and remodeling proteins to the host erythrocyte. Science.

[B78] Hiller NL, Bhattacharjee S, van Ooij C, Liolios K, Harrison T, Lopez-Estrano C, Haldar K (2004). A host-targeting signal in virulence proteins reveals a secretome in malarial infection. Science.

[B79] Castells E, Casacuberta JM (2007). Signalling through kinase-defective domains: the prevalence of atypical receptor-like kinases in plants. J Exp Bot.

[B80] Ventura JJ, Nebreda AR (2006). Protein kinases and phosphatases as therapeutic targets in cancer. Clin Transl Oncol.

[B81] Easty D, Gallagher W, Bennett DC (2006). Protein tyrosine phosphatases, new targets for cancer therapy. Curr Cancer Drug Targets.

[B82] Finn RD, Mistry J, Schuster-Bockler B, Griffiths-Jones S, Hollich V, Lassmann T, Moxon S, Marshall M, Khanna A, Durbin R (2006). Pfam: clans, web tools and services. Nucleic Acids Res.

[B83] Thompson JD, Higgins DG, Gibson TJ (1994). CLUSTAL W: improving the sensitivity of progressive multiple sequence alignment through sequence weighting, position-specific gap penalties and weight matrix choice. Nucleic Acids Res.

[B84] Notredame C, Higgins DG, Heringa J (2000). T-Coffee: A novel method for fast and accurate multiple sequence alignment. J Mol Biol.

[B85] Altschul SF, Madden TL, Schaffer AA, Zhang J, Zhang Z, Miller W, Lipman DJ (1997). Gapped BLAST and PSI-BLAST: a new generation of protein database search programs. Nucleic Acids Res.

[B86] Enright AJ, Van Dongen S, Ouzounis CA (2002). An efficient algorithm for large-scale detection of protein families. Nucleic Acids Res.

[B87] Huson DH, Bryant D (2006). Application of phylogenetic networks in evolutionary studies. Mol Biol Evol.

