# Supplementary material for: The protein-phosphatome of the human malaria parasite Plasmodium falciparum
Source: BMC Genomics. 2008 Sep 15;9:412. doi: 10.1186/1471-2164-9-412 (PMC2559854; doi:10.1186/1471-2164-9-412)
Supplement: Additional file 1 — List of PPP-conformant sequences. See legend within the file. [file 1471-2164-9-412-S1.doc]

| **1** | Q07099 | PP2A2_ARATH | Serine/threonine-protein phosphatase PP2A-2 c... |
| --- | --- | --- | --- |
| **2** | P48484 | PP14_ARATH | Serine/threonine-protein phosphatase PP1 isozy... |
| **3** | Q07100 | PP2A3_ARATH | Serine/threonine-protein phosphatase PP2A-3 c... |
| **4** | P48578 | PP2A3_ARATH | Serine/threonine-protein phosphatase PP2A-4 c... |
| **5** | Q84XU2 | Q84XU2_ARATH | Type 5 protein serine/threonine phosphatase ... |
| **6** | Q9M9W3 | Q9M9W3_ARATH | Putative serine/threonine protein phosphatas... |
| **7** | Q9LHE7 | Q9LHE7_ARATH | Phosphoprotein phosphatase (AT3g19980/MZE19_... |
| **8** | P48485 | PP15_ARATH | Serine/threonine-protein phosphatase PP1 isozy... |
| **9** | P48578 | PP2A4_ARATH | Serine/threonine-protein phosphatase PP2A-4 c... |
| **10** | Q8L7U5 | BSL1_ARATH | Serine/threonine-protein phosphatase BSL1 (EC ... |
| **11** | P48486 | PP16_ARATH | Serine/threonine-protein phosphatase PP1 isozy... |
| **12** | P48529 | PPX1_ARATH | Serine/threonine-protein phosphatase PP-X isoz... |
| **13** | Q9LEV0 | Q9LEV0_ARATH | Serine/threonine protein phosphatase-like pr... |
| **14** | O82734 | PP18_ARATH | Serine/threonine-protein phosphatase PP1 isozy... |
| **15** | O82734 | PP18_ARATH | Serine/threonine-protein phosphatase PP1 isozy... |
| **16** | O82733 | PP17_ARATH | Serine/threonine-protein phosphatase PP1 isozy... |
| **17** | O82733 | PP17_ARATH | Serine/threonine-protein phosphatase PP1 isozy... |
| **18** | P48528 | PPX2_ARATH | Serine/threonine-protein phosphatase PP-X isoz... |
| **19** | P48482 | PP12_ARATH | Serine/threonine-protein phosphatase PP1 isozy... |
| **20** | P48482 | PP12_ARATH | Serine/threonine-protein phosphatase PP1 isozy... |
| **21** | Q9LR78 | BSU1_ARATH | Serine/threonine-protein phosphatase BSU1 (EC ... |
| **22** | O49346 | O49346_ARATH | PP7 (EC 3.1.3.16) - Arabidopsis thaliana (Mo... |
| **23** | O49346 | O49346_ARATH | PP7 (EC 3.1.3.16) - Arabidopsis thaliana (Mo... |
| **24** | O49346 | O49346_ARATH | PP7 (EC 3.1.3.16) - Arabidopsis thaliana (Mo... |
| **25** | Q9LNG5 | Q9LNG5_ARATH | F21D18.16 - Arabidopsis thaliana (Mouse-ear ... |
| **26** | Q9SX52 | Q9SX52_ARATH | F14I3.5 protein (At1g50370) (Phosphoprotein ... |
| **27** | Q07099 | PP2A2_ARATH | Serine/threonine-protein phosphatase PP2A-2 c... |
| **28** | Q07099 | PP2A2_ARATH | Serine/threonine-protein phosphatase PP2A-2 c... |
| **29** | P48483 | PP13_ARATH | Serine/threonine-protein phosphatase PP1 isozy... |
| **30** | O04951 | PP2A5_ARATH | Serine/threonine-protein phosphatase PP2A-5 c... |
| **31** | Q9SHS7 | BSL3_ARATH | Serine/threonine-protein phosphatase BSL3 (EC ... |
| **32** | Q9SHS7 | BSL3_ARATH | Serine/threonine-protein phosphatase BSL3 (EC ... |
| **33** | P30366 | PP11_ARATH | Serine/threonine-protein phosphatase PP1 isozy... |
| **34** | Q54RH6 | Q54RH6_DICDI | Hypothetical protein - Dictyostelium discoid... |
| **35** | Q559Q6 | Q559Q6_DICDI | Protein phosphatase 6 catalytic subunit - Di... |
| **36** | O15757 | O15757_DICDI | Protein phosphatase type 1-like catalytic su... |
| **37** | Q54RD6 | Q54RD6_DICDI | Hypothetical protein - Dictyostelium discoid... |
| **38** | Q27560 | Q27560_DICDI | Calcineurin A - Dictyostelium discoideum (Sl... |
| **39** | Q559Z8 | Q559Z8_DICDI | Protein phosphatase 4 catalytic subunit - Di... |
| **40** | Q54G92 | Q54G92_DICDI | Protein phosphatase 2A catalytic subunit - D... |
| **41** | Q7R2V1 | Q7R2V1_GIALA | GLP_291_39422_40963 - Giardia lamblia ATCC 5... |
| **42** | Q7QXF4 | Q7QXF4_GIALA | GLP_14_36098_37354 - Giardia lamblia ATCC 50803 |
| **43** | Q7QZ40 | Q7QZ40_GIALA | GLP_464_14923_15879 - Giardia lamblia ATCC 5... |
| **44** | Q7R259 | Q7R259_GIALA | GLP_630_13393_12194 - Giardia lamblia ATCC 5... |
| **45** | Q7QZQ3 | Q7QZQ3_GIALA | GLP_680_19549_20568 - Giardia lamblia ATCC 5... |
| **46** | Q7QQ32 | Q7QQ32_GIALA | GLP_334_8230_9216 - Giardia lamblia ATCC 50803 |
| **47** | Q7QPH2 | Q7QPH2_GIALA | GLP_41_15091_14114 - Giardia lamblia ATCC 50803 |
| **48** | Q7R4Q0 | Q7R4Q0_GIALA | GLP_440_95652_94726 - Giardia lamblia ATCC 5... |
| **49** | Q7R4Q1 | Q7R4Q1_GIALA | GLP_440_94581_93649 - Giardia lamblia ATCC 5... |
| **50** | Q7R0G0 | Q7R0G0_GIALA | GLP_29_34461_37058 - Giardia lamblia ATCC 50803 |
| **51** | Q7R078 | Q7R078_GIALA | GLP_56_16828_19008 - Giardia lamblia ATCC 50803 |
| **52** | Q7QRV8 | Q7QRV8_GIALA | GLP_69_6397_7431 - Giardia lamblia ATCC 50803 |
| **53** | Q7QYH0 | Q7QYH0_GIALA | GLP_80_59047_59787 - Giardia lamblia ATCC 50803 |
| **54** | Q7QUF1 | Q7QUF1_GIALA | GLP_59_11104_12024 - Giardia lamblia ATCC 50803 |
| **55** | Q7R649 | Q7R649_GIALA | GLP_574_174171_172483 - Giardia lamblia ATCC... |
| **56** | P53041 | PPP5_HUMAN | Serine/threonine-protein phosphatase 5 (EC 3.1... |
| **57** | P62140 | PP1B_HUMAN | Serine/threonine-protein phosphatase PP1-beta ... |
| **58** | O14830 | PPE2_HUMAN | Serine/threonine-protein phosphatase with EF-h... |
| **59** | Q08209 | PP2BA_HUMAN | Serine/threonine-protein phosphatase 2B catal... |
| **60** | P67775 | PP2AA_HUMAN | Serine/threonine-protein phosphatase 2A catal... |
| **61** | P48454 | PP2BC_HUMAN | Serine/threonine-protein phosphatase 2B catal... |
| **62** | P62714 | PP2AB_HUMAN | Serine/threonine-protein phosphatase 2A catal... |
| **63** | O00743 | PPP6_HUMAN | Serine/threonine-protein phosphatase 6 (EC 3.1... |
| **64** | O14829 | PPE1_HUMAN | Serine/threonine-protein phosphatase with EF-h... |
| **65** | P16298 | PP2BB_HUMAN | Serine/threonine-protein phosphatase 2B catal... |
| **66** | P62136 | PP1A_HUMAN | Serine/threonine-protein phosphatase PP1-alpha... |
| **67** | P36873 | PP1G_HUMAN | Serine/threonine-protein phosphatase PP1-gamma... |
| **68** | P60510 | PP4C_HUMAN | Serine/threonine-protein phosphatase 4 catalyt... |
| **69** | Q8IKH5 | Q8IKH5_PLAF7 | Protein serine/threonine phosphatase - Plasm... |
| **70** | Q8ILV1 | Q8ILV1_PLAF7 | Serine/threonine protein phosphatase, putati... |
| **71** | Q8ILL9 | Q8ILL9_PLAF7 | PP1-like protein serine/threonine phosphatas... |
| **72** | O97259 | O97259_PLAF7 | Serine/threonine protein phosphatase (EC 3.1... |
| **73** | Q8IJL8 | Q8IJL8_PLAF7 | Erythrocyte membrane-associated antigen - Pl... |
| **74** | Q8IAM8 | Q8IAM8_PLAF7 | Serine/threonine protein phosphatase (EC 3.1... |
| **75** | Q8I2Q4 | Q8I2Q4_PLAF7 | Serine/threonine protein phosphatase (EC 3.1... |
| **76** | Q8I2N2 | Q8I2N2_PLAF7 | Serine/threonine protein phosphatase, putati... |
| **77** | Q8IDE7 | Q8IDE7_PLAF7 | Serine/threonine protein phosphatase pfPp5 (... |
| **78** | Q585I7 | Q585I7_9TRYP | Serine/threonine-protein phosphatase PP1, pu... |
| **79** | Q27786 | Q27786_9TRYP | Trypansoma brucei protein phosphatase 1 cata... |
| **80** | Q384L0 | Q384L0_9TRYP | Serine/threonine protein phosphatase catalyt... |
| **81** | Q38AW0 | Q38AW0_9TRYP | Serine/threonine protein phosphatase 2b cata... |
| **82** | Q4GYH2 | Q4GYH2_9TRYP | Ser/Thr protein phosphatase, putative - Tryp... |
| **83** | Q385R9 | Q385R9_9TRYP | Protein phosphotase, putative - Trypanosoma ... |
| **84** | Q382Y7 | Q382Y7_9TRYP | Protein phosphatase 4 catalytic subunit, put... |
| **85** | Q38AV2 | Q38AV2_9TRYP | Serine/threonine protein phosphatase, putati... |
| **86** | Q27786 | Q27786_9TRYP | Trypansoma brucei protein phosphatase 1 cata... |
| **87** | Q380Z0 | Q380Z0_9TRYP | Protein phosphatase 2A catalytic subunit (EC... |
| **88** | Q585I8 | Q585I8_9TRYP | Serine/threonine-protein phosphatase PP1, pu... |
| **89** | Q585I6 | Q585I6_9TRYP | Serine/threonine-protein phosphatase PP1, pu... |
| **90** | Q57Z11 | Q57Z11_9TRYP | Serine/threonine protein phosphatase, putati... |
| **91** | Q585J4 | Q585J4_9TRYP | Serine/threonine protein phosphatase PP1, pu... |
| **92** | Q382T3 | Q382T3_9TRYP | Serine/threonine protein phosphatase, putati... |
| **93** | Q583K6 | Q583K6_9TRYP | Serine/threonine protein phosphatase, putati... |
| **94** | Q585I9 | Q585I9_9TRYP | Serine/threonine-protein phosphatase PP1, pu... |
| **95** | Q38FZ2 | Q38FZ2_9TRYP | Serine/threonine protein phosphatase, putati... |
| **96** | Q57Z11 | Q57Z11_9TRYP | Serine/threonine protein phosphatase, putati... |
| **97** | Q388N2 | Q388N2_9TRYP | Serine/threonine protein phosphatase type 5 ... |
| **98** | Q57VP1 | Q57VP1_9TRYP | Hypothetical protein - Trypanosoma brucei |
| **99** | Q57XV7 | Q57XV7_9TRYP | Serine/threonine-protein phosphatase, putati... |
| **100** | Tp_166631 | _ |  |
| **101** | Tp_117876 | _ |  |
| **102** | Tp_125205 | _ |  |
| **103** | Tp_127188 | _ |  |
| **104** | Tp_127367 | _ |  |
| **105** | Tp_139225 | _ |  |
| **106** | Tp_147687 | _ |  |
| **107** | Tp_148198 | _ |  |
| **108** | Tp_150130 | _ |  |
| **109** | Q8RX38 | Q8RX38_ARATH | Truncated putative purple acid phosphatase -... |
| **110** | O48840 | O48840_ARATH | Putative purple acid phosphatase - Arabidops... |
| **111** | Q8RX38 | Q8RX38_ARATH | Truncated putative purple acid phosphatase -... |
| **112** | Q9SFU3 | Q9SFU3_ARATH | Putative purple acid phosphatase - Arabidops... |
| **113** | Q9LJU7 | Q9LJU7_ARATH | Purple acid phosphatase-like protein - Arabi... |
| **114** | Q9LMX4 | Q9LMX4_ARATH | F21F23.18 protein (Putative purple acid phos... |
| **115** | Q5MAU7 | Q5MAU7_ARATH | Putative purple acid phosphatase - Arabidops... |
| **116** | Q9LX83 | Q9LX83_ARATH | Purple acid phosphatase-like protein - Arabi... |
| **117** | Q9LXI7 | Q9LXI7_ARATH | Purple acid phosphatase-like protein (At3g52... |
| **118** | Q9LXI7 | Q9LXI7_ARATH | Purple acid phosphatase-like protein (At3g52... |
| **119** | Q9LXI4 | Q9LXI4_ARATH | Purple acid phosphatase-like protein - Arabi... |
| **120** | Q8S340 | Q8S340_ARATH | Purple acid phosphatase - Arabidopsis thalia... |
| **121** | Q9SVP2 | Q9SVP2_ARATH | Hypothetical protein F18A5.90 (Hypothetical ... |
| **122** | Q5MAV0 | Q5MAV0_ARATH | Putative purple acid phosphatase - Arabidops... |
| **123** | O23244 | O23244_ARATH | Purple acid phosphatase like protein (EC 3.1... |
| **124** | Q5MAU9 | Q5MAU9_ARATH | Putative purple acid phosphatase - Arabidops... |
| **125** | Q5MAU8 | Q5MAU8_ARATH | Putative purple acid phosphatase - Arabidops... |
| **126** | Q9C927 | Q9C927_ARATH | Putative purple acid phosphatase; 85474-9278... |
| **127** | Q9C510 | Q9C510_ARATH | Purple acid phosphatase, putative (At1g56360... |
| **128** | Q9ZQ81 | Q9ZQ81_ARATH | Putative purple acid phosphatase - Arabidops... |
| **129** | Q9SIV9 | Q9SIV9_ARATH | Putative purple acid phosphatase (At2g16430)... |
| **130** | Q9SIV9 | Q9SIV9_ARATH | Putative purple acid phosphatase (At2g16430)... |
| **131** | Q9SI18 | Q9SI18_ARATH | Putative purple acid phosphatase (At2g18130)... |
| **132** | Q38924 | PPAF_ARATH | Iron(III)-zinc(II) purple acid phosphatase pre... |
| **133** | Q54BS2 | Q54BS2_DICDI | Hypothetical protein - Dictyostelium discoid... |
| **134** | Q558S4 | Q558S4_DICDI | Hypothetical protein - Dictyostelium discoid... |
| **135** | Q54TC4 | Q54TC4_DICDI | Hypothetical protein - Dictyostelium discoid... |
| **136** | Q55F77 | Q55F77_DICDI | Hypothetical protein - Dictyostelium discoid... |
| **137** | Q54NC3 | Q54NC3_DICDI | Hypothetical protein - Dictyostelium discoid... |
| **138** | Q54SB4 | Q54SB4_DICDI | Hypothetical protein - Dictyostelium discoid... |
| **139** | Q6ZNF0 | Q6ZNF0_HUMAN | CDNA FLJ16165 fis, clone BRCOC2019841 - Homo... |
| **140** | Tp_148238 | _ |  |
| **141** | Q84LR5 | Q84LR5_ARATH | Hypothetical protein - Arabidopsis thaliana ... |
| **142** | Q84LR6 | Q84LR6_ARATH | Hypothetical protein - Arabidopsis thaliana ... |
| **143** | Q6GWE0 | Q6GWE0_ARATH | Putative purple acid phosphatase - Arabidops... |
| **144** | Q9LU72 | Q9LU72_ARATH | Emb CAB76911.1 (Putative purple acid phospha... |
| **145** | Q9FMK9 | Q9FMK9_ARATH | Emb CAB76911.1 (Putative purple acid phospha... |
| **146** | Q54R10 | Q54R10_DICDI | Hypothetical protein - Dictyostelium discoid... |
| **147** | Q54Q65 | Q54Q65_DICDI | Hypothetical protein - Dictyostelium discoid... |
| **148** | Q554Q5 | Q554Q5_DICDI | Hypothetical protein - Dictyostelium discoid... |
| **149** | Q54WW5 | Q54WW5_DICDI | Hypothetical protein - Dictyostelium discoid... |
| **150** | Q54YD1 | Q54YD1_DICDI | Hypothetical protein - Dictyostelium discoid... |
| **151** | Q54WW6 | Q54WW6_DICDI | Hypothetical protein - Dictyostelium discoid... |
| **152** | Q9SCX8 | Q9SCX8_ARATH | Acid phosphatase type 5 precursor (EC 3.1.3.... |
| **153** | Q8H129 | Q8H129_ARATH | Putative purple acid phosphatase - Arabidops... |
| **154** | Q8VYU7 | Q8VYU7_ARATH | At1g25230/F4F7_8 (Putative purple acid phosp... |
| **155** | Q8S341 | Q8S341_ARATH | Purple acid phosphatase (At2g01880) - Arabid... |
| **156** | Q3EC86 | Q3EC86_ARATH | Protein At2g01890 - Arabidopsis thaliana (Mo... |
| **157** | Q8VYZ2 | Q8VYZ2_ARATH | Putative purple acid phosphatase - Arabidops... |
| **158** | P13686 | PPA5_HUMAN | Tartrate-resistant acid phosphatase type 5 pre... |
| **159** | Tp_152188 | _ |  |
| **160** | Q94K01 | DBR1_ARATH | Lariat debranching enzyme (EC 3.1.-.-) (AtDBR1... |
| **161** | Q54VL9 | Q54VL9_DICDI | Hypothetical protein - Dictyostelium discoid... |
| **162** | Q9UK59 | DBR1_HUMAN | Lariat debranching enzyme (EC 3.1.-.-) - Homo ... |
| **163** | Q8IDT0 | Q8IDT0_PLAF7 | RNA lariat debranching enzyme, putative (EC ... |
| **164** | Q57ZT2 | Q57ZT2_9TRYP | Hypothetical protein - Trypanosoma brucei |
| **165** | Tp_139515 | _ |  |
| **166** | Q9XGM2 | MRE11_ARATH | Double-strand break repair protein MRE11 - Ar... |
| **167** | Q54BN2 | Q54BN2_DICDI | DNA repair exonuclease - Dictyostelium disco... |
| **168** | P49959 | MRE11_HUMAN | Double-strand break repair protein MRE11A (MR... |
| **169** | Q8I263 | Q8I263_PLAF7 | DNA repair exonuclease, putative - Plasmodiu... |
| **170** | Q586P4 | Q586P4_9TRYP | Endo/exonuclease Mre11 - Trypanosoma brucei |
| **171** | Tp_142334 | _ |  |
| **172** | Q55C09 | Q55C09_DICDI | Sphingomyelinase - Dictyostelium discoideum AX4 |
| **173** | Q54C16 | Q54C16_DICDI | Saposin B domain-containing protein - Dictyo... |
| **174** | Q54SR8 | Q54SR8_DICDI | Metallophosphoesterase domain-containing pro... |
| **175** | Q92484 | ASM3A_HUMAN | Acid sphingomyelinase-like phosphodiesterase ... |
| **176** | Q59EN6 | Q59EN6_HUMAN | Sphingomyelin phosphodiesterase 1, acid lyso... |
| **177** | Q92485 | ASM3B_HUMAN | Acid sphingomyelinase-like phosphodiesterase ... |
| **178** | Q9STT2 | Q9STT2_ARATH | Hypothetical protein T23J7.140 (Hypothetical... |
| **179** | Q9STT2 | Q9STT2_ARATH | Hypothetical protein T23J7.140 (Hypothetical... |
| **180** | Q54IF7 | Q54IF7_DICDI | Hypothetical protein - Dictyostelium discoid... |
| **181** | Q6IAH3 | Q6IAH3_HUMAN | VPS29 protein - Homo sapiens (Human) |
| **182** | Q8IM27 | Q8IM27_PLAF7 | Vacuolar protein sorting 29, putative - Plas... |
| **183** | Q9LPR3 | Q9LPR3_ARATH | F15H18.4 - Arabidopsis thaliana (Mouse-ear c... |
| **184** | Q8RY10 | Q8RY10_ARATH | At1g07010/F10K1_19 - Arabidopsis thaliana (M... |
| **185** | Q587C9 | Q587C9_9TRYP | Serine/threonine protein phosphatase, putati... |
| **186** | Tp_149252 | _ |  |
| **188** | Q583T9 | Q583T9_9TRYP | Serine/threonine protein phosphatase, putati... |
| **189** | Q57Y37 | Q57Y37_9TRYP | Serine/threonine protein phosphatase, putati... |
| **190** | Q57U41 | Q57U41_9TRYP | Diadenosine tetraphosphatase, putative (EC 3... |
| **191** | Q9T049 | Q9T049_ARATH | Hypothetical protein AT4g11800 - Arabidopsis... |
| **192** | O82748 | O82748_ARATH | Hypothetical protein F7H19.181 (Hypothetical... |
| **193** | Tp_102451 | _ |  |
| **194** | Q9SB68 | Q9SB68_ARATH | Hypothetical protein F22K18.70 (At4g24730) (... |
| **195** | Q9SB68 | Q9SB68_ARATH | Hypothetical protein F22K18.70 (At4g24730) (... |
| **196** | Tp_127209 | _ |  |
| **197** | Q9SR61 | Q9SR61_ARATH | T22K18.23 protein - Arabidopsis thaliana (Mo... |
| **198** | Q9SR62 | Q9SR62_ARATH | T22K18.22 protein (Hypothetical protein At3g... |
| **199** | Q54NN5 | Q54NN5_DICDI | Hypothetical protein - Dictyostelium discoid... |
| **200** | Q55D82 | Q55D82_DICDI | Hypothetical protein - Dictyostelium discoid... |
| **201** | Q86C19 | Q86C19_GIALA | Mre11 - Giardia lamblia (Giardia intestinalis) |
| **202** | Q86C19 | Q86C19_GIALA | Mre11 - Giardia lamblia (Giardia intestinalis) |
| **203** | Q9BRF8 | Q9BRF8_HUMAN | Hypothetical protein FLJ11151 (CSTP1) - Homo... |
| **204** | Q8IM55 | Q8IM55_PLAF7 | Acid phosphatase, putative - Plasmodium falc... |
| **205** | Q8IKE5 | Q8IKE5_PLAF7 | Hypothetical protein - Plasmodium falciparum... |
| **206** | Q8I5Y5 | Q8I5Y5_PLAF7 | Phosphoesterase, putative - Plasmodium falci... |
| **207** | Q0WVZ1 | Q0WVZ1_ARATH | Hypothetical protein At3g03300 - Arabidopsis... |
| **208** | Q9C8M6 | Q9C8M6_ARATH | Cell division control protein, putative; 159... |
| **209** | Q54PY8 | Q54PY8_DICDI | Hypothetical protein - Dictyostelium discoid... |
| **210** | Q54TW1 | Q54TW1_DICDI | Hypothetical protein - Dictyostelium discoid... |
| **211** | Q54KV3 | Q54KV3_DICDI | Hypothetical protein - Dictyostelium discoid... |
| **212** | Q54X09 | Q54X09_DICDI | Hypothetical protein - Dictyostelium discoid... |
| **213** | Q7R6P5 | Q7R6P5_GIALA | GLP_170_67460_69196 - Giardia lamblia ATCC 5... |
| **214** | Q7QSJ6 | Q7QSJ6_GIALA | GLP_195_803_2062 - Giardia lamblia ATCC 50803 |
| **215** | Q7QQX7 | Q7QQX7_GIALA | GLP_383_4433_5947 - Giardia lamblia ATCC 50803 |
| **216** | Q7R5V7 | Q7R5V7_GIALA | GLP_81_123727_121823 - Giardia lamblia ATCC ... |
| **217** | Q6ZRG1 | Q6ZRG1_HUMAN | CDNA FLJ46376 fis, clone TESTI4052430 - Homo... |
| **218** | Q6NZX3 | Q6NZX3_HUMAN | 5'-nucleotidase, ecto (CD73) - Homo sapiens ... |
| **219** | Q15777 | MPPD2_HUMAN | Metallophosphoesterase domain-containing prot... |
| **220** | Q53F39 | Q53F39_HUMAN | Metallophosphoesterase 1 variant (Fragment) ... |
| **221** | Q8IKJ1 | Q8IKJ1_PLAF7 | Hypothetical protein - Plasmodium falciparum... |
| **222** | Q583S8 | Q583S8_9TRYP | Serine/threonine protein phosphatase, putati... |
| **223** | Tp_110166 | _ |  |
| **224** | Tp_144982 | _ |  |

Additional File 1.

Sequences of the PPP group retrieved from the genomic databases using the PF00149 Pfam profile, with their database annotation. The organisms from which the sequences originate are colour-coded as follows: red, *P. falciparum* (Alveolates); green*, A. thaliana* (Plants); blue, *H. Sapiens* (Opisthokonts); turquoise, *G. lamblia* (Excavates); purple, *T. brucei* (Discicristates); black, *T. pseudonana* (Heterokonts); and magenta, *D. discoideum* (Amoebozoa). See text for details.
