# Supplementary material for: The protein-phosphatome of the human malaria parasite Plasmodium falciparum
Source: BMC Genomics. 2008 Sep 15;9:412. doi: 10.1186/1471-2164-9-412 (PMC2559854; doi:10.1186/1471-2164-9-412)
Supplement: Additional file 2 — List of PPP-conformant sequences. See legend within the file. [file 1471-2164-9-412-S2.doc]

| **1** | P93006 | P93006_ARATH | Hypothetical protein At2g33700 - Arabidopsis... |
| --- | --- | --- | --- |
| **2** | O64583 | O64583_ARATH | Hypothetical protein At2g34740 - Arabidopsis... |
| **3** | Q9XEE8 | Q9XEE8_ARATH | Protein phosphatase 2C (AthPP2C5) - Arabidop... |
| **4** | O22200 | O22200_ARATH | Hypothetical protein At2g40860 - Arabidopsis... |
| **5** | Q8W4L2 | Q8W4L2_ARATH | Hypothetical protein At3g02750; F13E7.31 - A... |
| **6** | Q9M9W9 | Q9M9W9_ARATH | Hypothetical protein F18C1.9 (AT3g05640/F18C... |
| **8** | Q7XJ53 | Q7XJ53_ARATH | At3g06270 (Hypothetical protein At3g06270) -... |
| **9** | P49598 | PP2C4_ARATH | Protein phosphatase 2C (EC 3.1.3.16) (PP2C) -... |
| **10** | Q9C7B3 | Q9C7B3_ARATH | Protein phosphatase 2C, putative; 16828-1828... |
| **11** | Q9LDA7 | Q9LDA7_ARATH | Protein phosphatase type 2C (Hypothetical pr... |
| **13** | Q9LUS8 | Q9LUS8_ARATH | Similarity to protein phosphatase-2c (At3g16... |
| **14** | Q9LRZ4 | Q9LRZ4_ARATH | Protein phosphatase-2C-like protein (Protein... |
| **16** | Q9LSN8 | Q9LSN8_ARATH | Protein phosphatase 2C-like protein - Arabid... |
| **17** | Q9LUU7 | Q9LUU7_ARATH | Protein phosphatase 2C-like protein (Protein... |
| **18** | Q9LW60 | Q9LW60_ARATH | Protein phosphatase 2C-like protein - Arabid... |
| **19** | Q3EAZ3 | Q3EAZ3_ARATH | Protein At3g27140 - Arabidopsis thaliana (Mo... |
| **20** | Q9SD12 | Q9SD12_ARATH | Protein phosphatase 2C-like protein - Arabid... |
| **21** | Q3EAM0 | Q3EAM0_ARATH | Protein At3g51370 - Arabidopsis thaliana (Mo... |
| **22** | Q9SD02 | Q9SD02_ARATH | Protein phosphatase 2C-like protein (At3g514... |
| **23** | Q94CL8 | Q94CL8_ARATH | Ser/Thr protein phosphatase 2C (Protein phos... |
| **24** | Q9M2W1 | Q9M2W1_ARATH | Protein phosphatase 2C-like protein - Arabid... |
| **25** | Q9SA22 | Q9SA22_ARATH | F3O9.3 protein - Arabidopsis thaliana (Mouse... |
| **26** | Q3EAF9 | Q3EAF9_ARATH | Protein At3g62260 - Arabidopsis thaliana (Mo... |
| **28** | Q9M1V8 | Q9M1V8_ARATH | Hypothetical protein F16M2_170 - Arabidopsis... |
| **29** | Q9M1V6 | Q9M1V6_ARATH | Hypothetical protein F16M2_190 - Arabidopsis... |
| **30** | Q8GY60 | Q8GY60_ARATH | Hypothetical protein (At4g03415) - Arabidops... |
| **31** | Q9SUF4 | Q9SUF4_ARATH | Hypothetical protein AT4g08260 - Arabidopsis... |
| **32** | Q8GWS8 | Q8GWS8_ARATH | Hypothetical protein At1g17550/F1L3_32 - Ara... |
| **33** | Q9T010 | Q9T010_ARATH | Hypothetical protein AT4g11040 - Arabidopsis... |
| **34** | Q9LMT1 | Q9LMT1_ARATH | T10F20.4 protein - Arabidopsis thaliana (Mou... |
| **35** | P49597 | PP2C1_ARATH | Protein phosphatase 2C ABI1 (EC 3.1.3.16) (PP... |
| **36** | P49599 | PP2C3_ARATH | Protein phosphatase 2C PPH1 (EC 3.1.3.16) (PP... |
| **39** | O49449 | O49449_ARATH | Protein phosphatase 2C-like protein - Arabid... |
| **40** | Q8RXV3 | Q8RXV3_ARATH | Hypothetical protein At4g31750 - Arabidopsis... |
| **41** | Q9SZ53 | Q9SZ53_ARATH | Protein phosphatase 2C-like protein (AT4g318... |
| **43** | O82637 | O82637_ARATH | Hypothetical protein AT4g32950 - Arabidopsis... |
| **44** | O81760 | O81760_ARATH | Hypothetical protein F17I5.110 (Hypothetical... |
| **45** | Q5PNS9 | Q5PNS9_ARATH | At4g38520 - Arabidopsis thaliana (Mouse-ear ... |
| **47** | Q6NKS1 | Q6NKS1_ARATH | At5g01700 - Arabidopsis thaliana (Mouse-ear ... |
| **48** | Q501F9 | Q501F9_ARATH | At5g02760 - Arabidopsis thaliana (Mouse-ear ... |
| **49** | Q9FG32 | Q9FG32_ARATH | Protein phosphatase 2C-like - Arabidopsis th... |
| **50** | Q8LAY8 | Q8LAY8_ARATH | Protein phosphatase 2C-like protein (Hypothe... |
| **51** | Q8LFF8 | Q8LFF8_ARATH | Protein phosphatase type 2C, putative - Arab... |
| **52** | Q3ED74 | Q3ED74_ARATH | Protein At1g22280 - Arabidopsis thaliana (Mo... |
| **53** | P46014 | KAPP_ARATH | Kinase-associated protein phosphatase (EC 3.1.... |
| **54** | Q4PSE8 | Q4PSE8_ARATH | Hypothetical protein - Arabidopsis thaliana ... |
| **55** | Q9XGZ9 | Q9XGZ9_ARATH | T1N24.8 protein - Arabidopsis thaliana (Mous... |
| **56** | Q0WRB2 | Q0WRB2_ARATH | Protein phosphatase 2C like protein (At5g279... |
| **57** | Q8LD08 | Q8LD08_ARATH | Protein phosphatase-like - Arabidopsis thali... |
| **58** | Q9FG61 | Q9FG61_ARATH | Protein phosphatase-2C PP2C-like - Arabidops... |
| **59** | Q9FLI3 | Q9FLI3_ARATH | Protein phosphatase-2C; PP2C-like protein - ... |
| **60** | Q94AT1 | Q94AT1_ARATH | Hypothetical protein At5g53140 (Putative pho... |
| **61** | O04719 | PP2C2_ARATH | Protein phosphatase 2C ABI2 (EC 3.1.3.16) (PP... |
| **62** | Q9FIF5 | Q9FIF5_ARATH | Similarity to Ca/calmodulin-dependent protei... |
| **63** | Q9FKX4 | Q9FKX4_ARATH | Protein phosphatase 2C-like protein - Arabid... |
| **64** | Q8SBC2 | Q8SBC2_ARATH | Protein phosphatase 2C (Fragment) - Arabidop... |
| **65** | Q9S9Z7 | Q9S9Z7_ARATH | F21H2.4 protein (Hypothetical protein At1g34... |
| **66** | Q8VZN9 | Q8VZN9_ARATH | Hypothetical protein At1g43900 - Arabidopsis... |
| **67** | Q9FX08 | Q9FX08_ARATH | T3F24.2 protein (Hypothetical protein At1g47... |
| **68** | Q9LNF4 | Q9LNF4_ARATH | F21D18.27 (At1g48040) (Protein phosphatase-2... |
| **69** | Q9FXE4 | Q9FXE4_ARATH | F12A21.5 - Arabidopsis thaliana (Mouse-ear c... |
| **70** | Q9M9C6 | Q9M9C6_ARATH | Hypothetical protein T2E12.9 (Hypothetical p... |
| **71** | Q9CAJ0 | Q9CAJ0_ARATH | Protein phosphatase 2C (AtP2C-HA); 19519-176... |
| **72** | Q8RX37 | Q8RX37_ARATH | Hypothetical protein At1g07160 - Arabidopsis... |
| **73** | Q8L7I4 | Q8L7I4_ARATH | At1g78200/T11I11_14 - Arabidopsis thaliana (... |
| **75** | Q9LNW3 | Q9LNW3_ARATH | F22G5.22 (Putative phosphatase 2C) (Hypothet... |
| **76** | Q8RXZ4 | Q8RXZ4_ARATH | Hypothetical protein At1g79630 - Arabidopsis... |
| **77** | Q2V4C0 | Q2V4C0_ARATH | Protein At1g79630 - Arabidopsis thaliana (Mo... |
| **78** | Q9SL76 | Q9SL76_ARATH | Hypothetical protein At2g20050 - Arabidopsis... |
| **79** | Q9SIU8 | Q9SIU8_ARATH | Hypothetical protein At2g20630 (At2g20630/F2... |
| **81** | O80492 | O80492_ARATH | T12M4.15 protein (At1g09160) - Arabidopsis t... |
| **83** | O81716 | O81716_ARATH | Hypothetical protein At2g25070 (Protein phos... |
| **84** | Q9SLA1 | Q9SLA1_ARATH | Hypothetical protein At2g25620 (Unnknown pro... |
| **85** | Q9ZW21 | Q9ZW21_ARATH | Hypothetical protein At2g29380 - Arabidopsis... |
| **86** | O80871 | O80871_ARATH | Hypothetical protein At2g30020 (Hypothetical... |
| **87** | Q54WS9 | Q54WS9_DICDI | Hypothetical protein - Dictyostelium discoid... |
| **88** | Q54T01 | Q54T01_DICDI | Hypothetical protein - Dictyostelium discoid... |
| **89** | Q54GL8 | Q54GL8_DICDI | Hypothetical protein - Dictyostelium discoid... |
| **90** | Q55C81 | Q55C81_DICDI | Hypothetical protein - Dictyostelium discoid... |
| **91** | Q54Y00 | Q54Y00_DICDI | Hypothetical protein - Dictyostelium discoid... |
| **92** | Q552E7 | Q552E7_DICDI | Protein serine/threonine phosphatase - Dicty... |
| **94** | Q554K8 | Q554K8_DICDI | Hypothetical protein - Dictyostelium discoid... |
| **95** | Q86A16 | Q86A16_DICDI | Similar to Medicago sativa (Alfalfa). Protei... |
| **96** | Q54QE5 | Q54QE5_DICDI | Hypothetical protein - Dictyostelium discoid... |
| **97** | Q7QUZ4 | Q7QUZ4_GIALA | GLP_61_27568_25133 - Giardia lamblia ATCC 50803 |
| **98** | Q24983 | Q24983_GIALA | Protein phosphatase 2C homolog - Giardia lam... |
| **99** | Q7QVQ7 | Q7QVQ7_GIALA | GLP_302_47488_46331 - Giardia lamblia ATCC 5... |
| **100** | Q7R0Z0 | Q7R0Z0_GIALA | GLP_25_29072_27444 - Giardia lamblia ATCC 50803 |
| **101** | Q7QNR2 | Q7QNR2_GIALA | GLP_279_6181_4310 - Giardia lamblia ATCC 50803 |
| **102** | Q6DKJ7 | Q6DKJ7_HUMAN | Protein phosphatase 1J (PP2C domain containi... |
| **103** | Q8N819 | Q8N819_HUMAN | CDNA FLJ40125 fis, clone TESTI2010872, weakl... |
| **104** | O15355 | PP2CG_HUMAN | Protein phosphatase 2C isoform gamma (EC 3.1.... |
| **105** | O75688 | PP2CB_HUMAN | Protein phosphatase 2C isoform beta (EC 3.1.3... |
| **106** | Q9H0C8 | ILKAP_HUMAN | Integrin-linked kinase-associated serine/thre... |
| **107** | Q6IPC0 | Q6IPC0_HUMAN | Protein phosphatase 1F (PP2C domain containi... |
| **108** | Q15750 | TAB1_HUMAN | Mitogen-activated protein kinase kinase kinase... |
| **109** | Q96MI6 | PP2CE_HUMAN | Protein phosphatase 2C isoform eta (EC 3.1.3.... |
| **110** | Q5SGD2 | PP2CL_HUMAN | Protein phosphatase 2C isoform epsilon (EC 3.... |
| **111** | Q8N3J5 | PP2CK_HUMAN | Protein phosphatase 2C isoform kappa (EC 3.1.... |
| **112** | Q9P0J1 | PDP1_HUMAN | [Pyruvate dehydrogenase [lipoamide]]-phosphata... |
| **113** | Q9ULR3 | Q9ULR3_HUMAN | Protein phosphatase 1H (Fragment) - Homo sap... |
| **114** | P35813 | PP2CA_HUMAN | Protein phosphatase 2C isoform alpha (EC 3.1.... |
| **115** | Q9P2J9 | PDP2_HUMAN | [Pyruvate dehydrogenase [lipoamide]]-phosphata... |
| **116** | Q6ZVD8 | PHLPL_HUMAN | PH domain leucine-rich repeat protein phospha... |
| **117** | Q8WY54 | Q8WY54_HUMAN | Protein phosphatase 1E (Partner of PIX 1) - ... |
| **118** | O15297 | PP2CD_HUMAN | Protein phosphatase 2C isoform delta (EC 3.1.... |
| **119** | O60346 | PHLPP_HUMAN | PH domain leucine-rich repeat-containing prot... |
| **120** | Q8IEM2 | Q8IEM2_PLAF7 | Protein phosphatase 2c-like protein, putativ... |
| **121** | Q8I4U1 | Q8I4U1_PLAF7 | Hypothetical protein - Plasmodium falciparum... |
| **122** | Q8IKS9 | Q8IKS9_PLAF7 | Protein phosphatase 2C, putative [PF14_0523] [Pl… |
| **123** | Q9U0I5 | Q9U0I5_PLAF7 | Protein phosphatase 2C (EC 3.1.3.16) - Plasm... |
| **124** | Q8I3Q9 | Q8I3Q9_PLAF7 | Protein phosphatase 2c, putative - Plasmodiu... |
| **125** | Q8II13 | Q8II13_PLAF7 | Hypothetical protein - Plasmodium falciparum... |
| **126** | Q8IHY0 | Q8IHY0_PLAF7 | Protein phosphatase 2C - Plasmodium falcipar... |
| **127** | Q8IAT8 | Q8IAT8_PLAF7 | Hypothetical protein MAL8P1.109 - Plasmodium... |
| **128** | Q8IAU0 | Q8IAU0_PLAF7 | Protein phosphatase, putative - Plasmodium f... |
| **129** | Q583D6 | Q583D6_9TRYP | Protein phosphatase 2C, putative (EC 3.1.3.1... |
| **130** | Q387H4 | Q387H4_9TRYP | Protein phosphatase 2C, putative (EC 3.1.3.1... |
| **131** | Q57UN2 | Q57UN2_9TRYP | Protein phosphatase 2C, putative (EC 3.1.3.1... |
| **132** | Q585N1 | Q585N1_9TRYP | Protein phosphatase 2C, putative (EC 3.1.3.1... |
| **133** | Q586U3 | Q586U3_9TRYP | Protein phosphatase 2C, putative (EC 3.1.3.1... |
| **134** | Q381U4 | Q381U4_9TRYP | Protein phosphatase 2C, putative (EC 3.1.3.1... |
| **135** | Q583I2 | Q583I2_9TRYP | Protein phosphatase 2C, putative (EC 3.1.3.1... |
| **136** | Q57V97 | Q57V97_9TRYP | Protein phosphatase 2C, putative (EC 3.1.3.1... |
| **137** | Q38BA8 | Q38BA8_9TRYP | Protein phosphatase 2C-like, putative (EC 3.... |
| **138** | Tp_165726 | _ |  |
| **139** | Tp_110630 | _ |  |
| **140** | Tp_111933 | _ |  |
| **141** | Tp_113231 | _ |  |
| **142** | Tp_115419 | _ |  |
| **143** | Tp_121256 | _ |  |
| **144** | Tp_124205 | _ |  |
| **145** | Tp_124499 | _ |  |
| **146** | Tp_126708 | _ |  |
| **147** | Tp_130483 | _ |  |
| **148** | Tp_131766 | _ |  |
| **149** | Tp_134639 | _ |  |
| **150** | Tp_136724 | _ |  |
| **151** | Tp_143246 | _ |  |
| **152** | Tp_143986 | _ |  |
| **153** | Tp_147972 | _ |  |
| **154** | Tp_154487 | _ |  |
| **155** | Tp_158708 | _ |  |
| **156** | Q8GY02 | Q8GY02_ARATH | Hypothetical protein At4g16580/dl4315c (At4g... |
| **157** | Q93V88 | Q93V88_ARATH | Hypothetical protein At4g33500 - Arabidopsis... |
| **158** | Q3E849 | Q3E849_ARATH | Protein At5g66720 - Arabidopsis thaliana (Mo... |
| **159** | O64730 | O64730_ARATH | Expressed protein (Hypothetical protein At2g... |
| **160** | Q54JD8 | Q54JD8_DICDI | Hypothetical protein - Dictyostelium discoid... |
| **162** | Q8NI37 | Q8NI37_HUMAN | T-cell activation protein phosphatase 2C (PT... |
| **163** | Q8IJU9 | Q8IJU9_PLAF7 | Hypothetical protein - Plasmodium falciparum... |
| **164** | Q585I2 | Q585I2_9TRYP | Protein phosphatase 2C, putative - Trypanoso... |
| **165** | Q57Z96 | Q57Z96_9TRYP | Protein phosphatase 2C, putative - Trypanoso... |
| **166** | Q386X6 | Q386X6_9TRYP | Protein phosphatase 2C, putative - Trypanoso... |
| **167** | Tp_119725 | _ |  |
| **168** | Tp_132369 | _ |  |
| **169** | O82302 | O82302_ARATH | Hypothetical protein At2g35350 - Arabidopsis... |
| **170** | Q8RWN7 | Q8RWN7_ARATH | Hypothetical protein At2g46920 - Arabidopsis... |
| **172** | Q9SR24 | Q9SR24_ARATH | F3L24.29 protein - Arabidopsis thaliana (Mou... |
| **173** | Q9LZ86 | Q9LZ86_ARATH | Hypothetical protein T1E22_160 - Arabidopsis... |
| **174** | Q9LQN6 | Q9LQN6_ARATH | F24B9.31 protein - Arabidopsis thaliana (Mou... |
| **175** | Q9ZV25 | Q9ZV25_ARATH | Hypothetical protein At2g28890 - Arabidopsis... |
| **176** | Q9SR68 | Q9SR68_ARATH | Putative uridylate kinase - Arabidopsis thal... |
| **177** | Q7QRJ6 | Q7QRJ6_GIALA | GLP_290_190_2160 - Giardia lamblia ATCC 50803 |
| **178** | Q7R0U8 | Q7R0U8_GIALA | GLP_186_20267_18732 - Giardia lamblia ATCC 5... |
| **179** | Q38B45 | Q38B45_9TRYP | Protein phosphatase 2C, putative (EC 3.1.3.1... |
| **180** | Q586P1 | Q586P1_9TRYP | Beta prime COP protein - Trypanosoma brucei |

Additional file 2.

Sequences of the PPM group retrieved from the genomic databases using the PF00481 Pfam profile, with their database annotation. The organisms from which the sequences originate are colour-coded as follows: red, *P. falciparum* (Alveolates); green*, A. thaliana* (Plants); blue, *H. Sapiens* (Opisthokonts); turquoise, *G. lamblia* (Excavates); purple, *T. brucei* (Discicristates); black, *T. pseudonana* (Heterokonts); and magenta, *D. discoideum* (Amoebozoa). See text for details.
