# Supplementary material for: The protein-phosphatome of the human malaria parasite Plasmodium falciparum
Source: BMC Genomics. 2008 Sep 15;9:412. doi: 10.1186/1471-2164-9-412 (PMC2559854; doi:10.1186/1471-2164-9-412)
Supplement: Additional file 3 — List of PTP-conformant sequences. See legend within the file. [file 1471-2164-9-412-S3.doc]

| **1** | Q9ZQP1 | Q9ZQP1_ARATH | Expressed protein (Hypothetical protein At2g... |
| --- | --- | --- | --- |
| **2** | Q9M8K7 | Q9M8K7_ARATH | Putative dual-specificity protein phosphatas... |
| **3** | Q8LDW2 | Q8LDW2_ARATH | Putative dual-specificity protein phosphatas... |
| **4** | Q9ZR37 | Q9ZR37_ARATH | DsPTP1 protein - Arabidopsis thaliana (Mouse... |
| **5** | Q8L613 | Q8L613_ARATH | Hypothetical protein At5g23720 - Arabidopsis... |
| **6** | Q75QN6 | Q75QN6_ARATH | PROPYZAMIDE-HTPERSENSITIVE 1 - Arabidopsis t... |
| **7** | Q9FJU7 | Q9FJU7_ARATH | Similarity to unknown protein - Arabidopsis ... |
| **8** | Q84JU4 | Q84JU4_ARATH | Hypothetical protein At2g04550 (Dual-specifi... |
| **9** | Q3EC68 | Q3EC68_ARATH | Protein At2g04550 - Arabidopsis thaliana (Mo... |
| **10** | Q84JU4 | Q84JU4_ARATH | Hypothetical protein At2g04550 (Dual-specifi... |
| **11** | Q54J65 | Q54J65_DICDI | MAP kinase phosphatase - Dictyostelium disco... |
| **12** | Q55E39 | Q55E39_DICDI | Hypothetical protein - Dictyostelium discoid... |
| **13** | Q55CS7 | Q55CS7_DICDI | Hypothetical protein - Dictyostelium discoid... |
| **14** | Q55BI8 | Q55BI8_DICDI | Hypothetical protein - Dictyostelium discoid... |
| **15** | Q86JT3 | Q86JT3_DICDI | Similar to dual-specificity protein phosphat... |
| **16** | Q8T813 | Q8T813_DICDI | Similar to Dictyostelium discoideum (Slime m... |
| **17** | Q86JT3 | Q86JT3_DICDI | Similar to dual-specificity protein phosphat... |
| **18** | Q54T76 | Q54T76_DICDI | Hypothetical protein - Dictyostelium discoid... |
| **19** | Q86B02 | Q86B02_DICDI | Similar to Dictyostelium discoideum (Slime m... |
| **20** | Q55CS8 | Q55CS8_DICDI | Hypothetical protein - Dictyostelium discoid... |
| **21** | Q54Y32 | Q54Y32_DICDI | Hypothetical protein - Dictyostelium discoid... |
| **22** | Q54CK7 | Q54CK7_DICDI | Hypothetical protein - Dictyostelium discoid... |
| **23** | Q54R42 | Q54R42_DICDI | Hypothetical protein - Dictyostelium discoid... |
| **24** | Q54N84 | Q54N84_DICDI | Hypothetical protein - Dictyostelium discoid... |
| **25** | Q7R240 | Q7R240_GIALA | GLP_630_38763_38242 - Giardia lamblia ATCC 5... |
| **26** | Q7QTA9 | Q7QTA9_GIALA | GLP_15_17049_19172 - Giardia lamblia ATCC 50803 |
| **27** | Q7QW29 | Q7QW29_GIALA | GLP_457_46033_48018 - Giardia lamblia ATCC 5... |
| **28** | Q05923 | DUS2_HUMAN | Dual specificity protein phosphatase 2 (EC 3.1... |
| **29** | Q8WTR2 | DUS19_HUMAN | Dual specificity protein phosphatase 19 (EC 3... |
| **30** | Q4G0W2 | Q4G0W2_HUMAN | DUSP28 protein - Homo sapiens (Human) |
| **31** | Q9H1R2 | DUS15_HUMAN | Dual specificity protein phosphatase 15 (EC 3... |
| **32** | Q8NEJ0 | DUS18_HUMAN | Dual specificity protein phosphatase 18 (EC 3... |
| **33** | Q16829 | DUS7_HUMAN | Dual specificity protein phosphatase 7 (EC 3.1... |
| **34** | Q9UNI6 | DUS12_HUMAN | Dual specificity protein phosphatase 12 (EC 3... |
| **35** | Q5VZP5 | Q5VZP5_HUMAN | Dual specificity phosphatase 27 - Homo sapie... |
| **36** | P28562 | DUS1_HUMAN | Dual specificity protein phosphatase 1 (EC 3.1... |
| **37** | Q9NRW4 | DUS22_HUMAN | Dual specificity protein phosphatase 22 (EC 3... |
| **38** | Q9Y6W6 | DUS10_HUMAN | Dual specificity protein phosphatase 10 (EC 3... |
| **39** | Q13115 | DUS4_HUMAN | Dual specificity protein phosphatase 4 (EC 3.1... |
| **40** | Q9BV47 | Q9BV47_HUMAN | Dual specificity phosphatase 26 (Putative) (... |
| **41** | Q9H596 | DUS21_HUMAN | Dual specificity protein phosphatase 21 (EC 3... |
| **42** | Q99956 | DUS9_HUMAN | Dual specificity protein phosphatase 9 (EC 3.1... |
| **43** | Q86SS8 | Q86SS8_HUMAN | Dual specificity phosphatase 8 - Homo sapien... |
| **44** | Q68J44 | Q68J44_HUMAN | Dual specificity phosphatase and pro isomera... |
| **45** | Q9UII6 | DUS13_HUMAN | Dual specificity protein phosphatase 13 (EC 3... |
| **46** | Q5T603 | Q5T603_HUMAN | Dual specificity phosphatase 5 - Homo sapien... |
| **47** | Q86SS8 | Q86SS8_HUMAN | Dual specificity phosphatase 8 - Homo sapien... |
| **48** | Q8TE77 | SSH3_HUMAN | Protein phosphatase Slingshot homolog 3 (EC 3.... |
| **49** | Q9BY84 | DUS16_HUMAN | Dual specificity protein phosphatase 16 (EC 3... |
| **50** | Q16828 | DUS6_HUMAN | Dual specificity protein phosphatase 6 (EC 3.1... |
| **51** | Q8WYL5 | SSH1_HUMAN | Protein phosphatase Slingshot homolog 1 (EC 3.... |
| **52** | Q8WUJ0 | STYX_HUMAN | Serine/threonine/tyrosine-interacting protein ... |
| **53** | Q76I76 | SSH2_HUMAN | Protein phosphatase Slingshot homolog 2 (EC 3.... |
| **54** | O95147 | DUS14_HUMAN | Dual specificity protein phosphatase 14 (EC 3... |
| **55** | P51452 | DUS3_HUMAN | Dual specificity protein phosphatase 3 (EC 3.1... |
| **56** | Q8IKS7 | Q8IKS7_PLAF7 | Hypothetical protein - Plasmodium falciparum... |
| **57** | O77334 | O77334_PLAF7 | Dual-specificity protein phosphatase, putati... |
| **58** | Q384M4 | Q384M4_9TRYP | Dual specificity protein phosphatase, putati... |
| **59** | Q586U9 | Q586U9_9TRYP | Dual specificity protein phosphatase, putati... |
| **60** | Q584E1 | Q584E1_9TRYP | Dual specificity protein phosphatase, putati... |
| **61** | Q38E03 | Q38E03_9TRYP | Dual specificity protein phosphatase, putati... |
| **62** | Q586S8 | Q586S8_9TRYP | Dual specificity protein phosphatase, putati... |
| **63** | Q387M2 | Q387M2_9TRYP | Hypothetical protein - Trypanosoma brucei |
| **64** | Q384T3 | Q384T3_9TRYP | Phopshatase, putative - Trypanosoma brucei |
| **65** | Q389R1 | Q389R1_9TRYP | Dual specificity protein phosphatase, putati... |
| **66** | Q57UA4 | Q57UA4_9TRYP | Dual-specificity protein phosphatase, putati... |
| **67** | Q38C79 | Q38C79_9TRYP | Dual specificity protein phosphatase, putati... |
| **68** | Tp_103798 | _ |  |
| **69** | Tp_128948 | _ |  |
| **70** | Tp_98866 | _ |  |
| **71** | Tp_140676 | _ |  |
| **72** | Tp_157203 | _ |  |
| **73** | Tp_157244 | _ |  |
| **74** | O65190 | O65190_ARATH | Protein tyrosine phosphatase 1 - Arabidopsis... |
| **75** | Q3ECD9 | Q3ECD9_ARATH | Protein At1g71860 - Arabidopsis thaliana (Mo... |
| **76** | P54637 | PTP3_DICDI | Tyrosine-protein phosphatase 3 (EC 3.1.3.48) (... |
| **77** | Q86AJ9 | Q86AJ9_DICDI | Similar to Dictyostelium discoideum (Slime m... |
| **78** | P34138 | PTP2_DICDI | Tyrosine-protein phosphatase 2 (EC 3.1.3.48) (... |
| **79** | P34137 | PTP1_DICDI | Tyrosine-protein phosphatase 1 (EC 3.1.3.48) (... |
| **81** | Q9UM81 | Q9UM81_HUMAN | PTPsigma-(Brain) precursor - Homo sapiens (H... |
| **82** | Q9Y2R2 | PTN22_HUMAN | Tyrosine-protein phosphatase non-receptor typ... |
| **83** | Q15426 | Q15426_HUMAN | Protein-tyrosine phosphatase receptor type H... |
| **84** | P29074 | PTN4_HUMAN | Tyrosine-protein phosphatase non-receptor type... |
| **85** | Q99952 | PTN18_HUMAN | Tyrosine-protein phosphatase non-receptor typ... |
| **86** | Q16849 | PTPRN_HUMAN | Receptor-type tyrosine-protein phosphatase-li... |
| **87** | O14522 | PTPRT_HUMAN | Receptor-type tyrosine-protein phosphatase T ... |
| **88** | P18031 | PTN1_HUMAN | Tyrosine-protein phosphatase non-receptor type... |
| **89** | Q9H3S7 | PTN23_HUMAN | Tyrosine-protein phosphatase non-receptor typ... |
| **90** | P23470 | PTPRG_HUMAN | Receptor-type tyrosine-protein phosphatase ga... |
| **91** | Q06124 | PTN11_HUMAN | Tyrosine-protein phosphatase non-receptor typ... |
| **92** | Q12923 | PTN13_HUMAN | Tyrosine-protein phosphatase non-receptor typ... |
| **93** | P08575 | CD45_HUMAN | Leukocyte common antigen precursor (EC 3.1.3.4... |
| **94** | P35236 | PTN7_HUMAN | Tyrosine-protein phosphatase non-receptor type... |
| **95** | Q15262 | PTPRK_HUMAN | Receptor-type tyrosine-protein phosphatase ka... |
| **96** | Q05209 | PTN12_HUMAN | Tyrosine-protein phosphatase non-receptor typ... |
| **97** | P23471 | PTPRZ_HUMAN | Receptor-type tyrosine-protein phosphatase ze... |
| **98** | Q15678 | PTN14_HUMAN | Tyrosine-protein phosphatase non-receptor typ... |
| **99** | Q92932 | PTPR2_HUMAN | Receptor-type tyrosine-protein phosphatase N2... |
| **100** | Q06124 | PTN11_HUMAN | Tyrosine-protein phosphatase non-receptor typ... |
| **101** | P23468 | PTPRD_HUMAN | Receptor-type tyrosine-protein phosphatase de... |
| **102** | P26045 | PTN3_HUMAN | Tyrosine-protein phosphatase non-receptor type... |
| **103** | Q06124 | PTN11_HUMAN | Tyrosine-protein phosphatase non-receptor typ... |
| **104** | Q4JDL8 | Q4JDL8_HUMAN | Non-receptor protein tyrosine phosphatase 20... |
| **105** | Q4JDL8 | Q4JDL8_HUMAN | Non-receptor protein tyrosine phosphatase 20... |
| **106** | P23469 | PTPRE_HUMAN | Receptor-type tyrosine-protein phosphatase ep... |
| **107** | Q6P1Z2 | Q6P1Z2_HUMAN | Protein tyrosine phosphatase, non-receptor t... |
| **108** | Q12913 | PTPRJ_HUMAN | Receptor-type tyrosine-protein phosphatase et... |
| **109** | Q5VSZ4 | Q5VSZ4_HUMAN | Protein tyrosine phosphatase, receptor type,... |
| **110** | P29350 | PTN6_HUMAN | Tyrosine-protein phosphatase non-receptor type... |
| **111** | Q16827 | PTPRO_HUMAN | Receptor-type tyrosine-protein phosphatase O ... |
| **112** | P23467 | PTPRB_HUMAN | Receptor-type tyrosine-protein phosphatase be... |
| **113** | Q05B41 | Q05B41_HUMAN | PTPRR protein - Homo sapiens (Human) |
| **114** | Q9UMZ3 | Q9UMZ3_HUMAN | Protein-tyrosine phosphatase receptor type Q... |
| **115** | Q06124 | PTN11_HUMAN | Tyrosine-protein phosphatase non-receptor typ... |
| **116** | P10586 | PTPRF_HUMAN | Receptor-type tyrosine-protein phosphatase F ... |
| **117** | Q16825 | PTN21_HUMAN | Tyrosine-protein phosphatase non-receptor typ... |
| **118** | P43378 | PTN9_HUMAN | Tyrosine-protein phosphatase non-receptor type... |
| **119** | P28827 | PTPRM_HUMAN | Receptor-type tyrosine-protein phosphatase mu... |
| **120** | P17706 | PTN2_HUMAN | Tyrosine-protein phosphatase non-receptor type... |
| **121** | Q38AT7 | Q38AT7_9TRYP | Tyrosine specific protein phosphatase, putat... |
| **123** | Q558S1 | Q558S1_DICDI | Hypothetical protein - Dictyostelium discoid... |
| **124** | Q558S1 | Q558S1_DICDI | Hypothetical protein - Dictyostelium discoid... |
| **125** | Q7QWV9 | Q7QWV9_GIALA | GLP_203_38772_36940 - Giardia lamblia ATCC 5... |
| **126** | Q9BVJ7 | DUS23_HUMAN | Dual specificity protein phosphatase 23 (EC 3... |
| **127** | Q93096 | TP4A1_HUMAN | Protein tyrosine phosphatase type IVA protein... |
| **128** | Q93096 | TP4A1_HUMAN | Protein tyrosine phosphatase type IVA protein... |
| **129** | Q6NUS3 | Q6NUS3_HUMAN | CDC14 cell division cycle 14 homolog C (Frag... |
| **130** | O75365 | TP4A3_HUMAN | Protein tyrosine phosphatase type IVA protein... |
| **131** | A2A3K4 | A2A3K4_HUMAN | Protein tyrosine phosphatase domain containi... |
| **132** | O60729 | CC14B_HUMAN | Dual specificity protein phosphatase CDC14B (... |
| **133** | Q12974 | TP4A2_HUMAN | Protein tyrosine phosphatase type IVA protein... |
| **134** | Q5VUH8 | Q5VUH8_HUMAN | CDC14 cell division cycle 14 homolog A (S. c... |
| **135** | Q8IIN1 | Q8IIN1_PLAF7 | Protein tyrosine phosphatase, putative - Pla... |
| **136** | Q382T8 | Q382T8_9TRYP | Tyrosine phosphatase, putative - Trypanosoma... |
| **137** | Q57YH9 | Q57YH9_9TRYP | Protein tyrosine phosphatase, putative (EC 3... |
| **138** | Tp_171977 | _ |  |
| **139** | Tp_139348 | _ |  |
| **140** | Q84MD6 | Q84MD6_ARATH | At2g32960 (Hypothetical protein At2g32960) -... |
| **141** | Q9M8S1 | Q9M8S1_ARATH | F13E7.26 protein (At3g02800) - Arabidopsis t... |
| **142** | Q940L5 | Q940L5_ARATH | AT4g03960/T24M8_4 (Contains similarity to C3... |
| **143** | Q9FFD7 | Q9FFD7_ARATH | Similarity to tyrosine phosphatase (At5g1648... |
| **144** | Q9ZVN4 | Y1500_ARATH | Probable tyrosine-protein phosphatase At1g050... |
| **145** | Q1ZXG8 | Q1ZXG8_DICDI | Putative tyrosine phosphatase - Dictyosteliu... |
| **146** | Q54MJ2 | Q54MJ2_DICDI | Hypothetical protein - Dictyostelium discoid... |
| **147** | Q54VX9 | Q54VX9_DICDI | Hypothetical protein - Dictyostelium discoid... |
| **148** | Q54MS1 | Q54MS1_DICDI | Hypothetical protein - Dictyostelium discoid... |
| **149** | Q38EH9 | Q38EH9_9TRYP | Hypothetical protein - Trypanosoma brucei |
| **150** | Q387J3 | Q387J3_9TRYP | Hypothetical protein - Trypanosoma brucei |
| **151** | Q38EH9 | Q38EH9_9TRYP | Hypothetical protein - Trypanosoma brucei |
| **152** | Q382B1 | Q382B1_9TRYP | Hypothetical protein - Trypanosoma brucei |
| **153** | Q9SS77 | Q9SS77_ARATH | Putative mRNA capping enzyme, RNA guanylyltr... |
| **154** | Q9SS77 | Q9SS77_ARATH | Putative mRNA capping enzyme, RNA guanylyltr... |
| **155** | Q8GSD7 | Q8GSD7_ARATH | MRNA capping enzyme-like protein - Arabidops... |
| **156** | Q9S9L3 | Q9S9L3_ARATH | F26C17.2 protein - Arabidopsis thaliana (Mou... |
| **157** | O75319 | DUS11_HUMAN | RNA/RNP complex-1-interacting phosphatase (EC... |
| **158** | O60942 | MCE1_HUMAN | mRNA capping enzyme (HCE) (HCAP1) [Includes: P... |
| **159** | Q9FLZ5 | Q9FLZ5_ARATH | Similarity to protein-tyrosine phosphatase (... |
| **160** | Q553B4 | Q553B4_DICDI | Hypothetical protein - Dictyostelium discoid... |
| **161** | Q54CH3 | Q54CH3_DICDI | Hypothetical protein - Dictyostelium discoid... |
| **162** | Q8T9S7 | Q8T9S7_DICDI | Pten 3-phosphoinositide phosphatase alpha - ... |
| **163** | Q54JL7 | Q54JL7_DICDI | Putative countin receptor Cnr15 - Dictyostel... |
| **164** | Q9SSA3 | Q9SSA3_ARATH | F4P13.6 protein - Arabidopsis thaliana (Mous... |
| **165** | Q9SRK5 | Q9SRK5_ARATH | F9F8.24 protein (Hypothetical protein At3g10... |
| **166** | Q9FEB5 | Q9FEB5_ARATH | PTPKIS1 protein - Arabidopsis thaliana (Mous... |
| **167** | Q559B2 | Q559B2_DICDI | Hypothetical protein PLIP - Dictyostelium di... |
| **168** | O95278 | EPM2A_HUMAN | Laforin (EC 3.1.3.48) (EC 3.1.3.16) (Lafora P... |
| **169** | Q16667 | CDKN3_HUMAN | Cyclin-dependent kinase inhibitor 3 (EC 3.1.3... |
| **170** | Q8II93 | Q8II93_PLAF7 | Hypothetical protein - Plasmodium falciparum... |
| **171** | Q382S4 | Q382S4_9TRYP | Hypothetical protein - Trypanosoma brucei |
| **172** | Tp_111465 | _ |  |

Additional file 3.

Sequences of the PTP group retrieved from the genomic databases using the PF00102, PF00782 and PF03162 Pfam profiles, with their database annotation. The organisms from which the sequences originate are colour-coded as follows: red, *P. falciparum* (Alveolates); green*, A. thaliana* (Plants); blue, *H. Sapiens* (Opisthokonts); turquoise, *G. lamblia* (Excavates); purple, *T. brucei* (Discicristates); black, *T. pseudonana* (Heterokonts); and magenta, *D. discoideum* (Amoebozoa). See text for details.
