# Supplementary material for: The protein-phosphatome of the human malaria parasite Plasmodium falciparum
Source: BMC Genomics. 2008 Sep 15;9:412. doi: 10.1186/1471-2164-9-412 (PMC2559854; doi:10.1186/1471-2164-9-412)
Supplement: Additional file 4 — List of NIF-conformant sequences. See legend within the file. [file 1471-2164-9-412-S4.doc]

| **1** | Q9LY49 | Q9LY49_ARATH | Hypothetical protein F27K19 140 (Hypothetica... |
| --- | --- | --- | --- |
| **2** | Q9SVV8 | Q9SVV8_ARATH | Hypothetical protein F15J5.110 (Hypothetical... |
| **3** | Q9LYI7 | Q9LYI7_ARATH | Hypothetical protein F14F18 30 (At5g11860) (... |
| **4** | Q9LYI7 | Q9LYI7_ARATH | Hypothetical protein F14F18 30 (At5g11860) (... |
| **5** | Q9FK73 | Q9FK73_ARATH | Arabidopsis thaliana genomic DNA, chromosome... |
| **6** | Q8GYV2 | Q8GYV2_ARATH | Hypothetical protein At5g46410/MPL12 21 - Ar... |
| **7** | Q9FXF4 | Q9FXF4_ARATH | F1N18.17 protein (At1g29770) - Arabidopsis t... |
| **8** | Q9FXF5 | Q9FXF5_ARATH | F1N18.16 protein - Arabidopsis thaliana (Mou... |
| **9** | Q8VYE2 | TIM50_ARATH | Import inner membrane translocase subunit TIM... |
| **10** | Q54M72 | Q54M72_DICDI | Hypothetical protein - Dictyostelium discoid... |
| **11** | Q54GB2 | Q54GB2_DICDI | Hypothetical protein - Dictyostelium discoid... |
| **12** | Q54M73 | Q54M73_DICDI | Hypothetical protein - Dictyostelium discoid... |
| **13** | Q54AK8 | Q54AK8_DICDI | Putative CTD phosphatase - Dictyostelium dis... |
| **14** | Q7QZ65 | Q7QZ65_GIALA | GLP 22 23484 22576 - Giardia lamblia ATCC 50803 |
| **15** | Q7R5U8 | Q7R5U8_GIALA | GLP 81 145711 146535 - Giardia lamblia ATCC ... |
| **16** | Q7QXW9 | Q7QXW9_GIALA | GLP 479 51161 49863 - Giardia lamblia ATCC 5... |
| **17** | Q7QQZ2 | Q7QQZ2_GIALA | GLP 442 14601 13606 - Giardia lamblia ATCC 5... |
| **18** | Q3ZCQ8 | TIM50_HUMAN | Import inner membrane translocase subunit TIM... |
| **19** | Q9GZU7 | CTDS1_HUMAN | Carboxy-terminal domain RNA polymerase II pol... |
| **20** | Q3ZTU0 | Q3ZTU0_HUMAN | HYA22 - Homo sapiens (HUMAN ) |
| **21** | Q53ZR2 | Q53ZR2_HUMAN | Small CTD phosphatase 2 - Homo sapiens (HUMAN ) |
| **22** | Q53ZR2 | Q53ZR2_HUMAN | Small CTD phosphatase 2 - Homo sapiens (HUMAN ) |
| **23** | Q8IYI9 | Q8IYI9_HUMAN | CTDSPL2 protein (Hypothetical protein) - Hom... |
| **24** | O95476 | O95476_HUMAN | Protein dullard homolog - Homo sapiens (HUMAN ) |
| **25** | Q8I3U9 | Q8I3U9_PLAF7 | Nif-like protein, putative - Plasmodium falc... |
| **26** | Q8IBI8 | Q8IBI8_PLAF7 | Hypothetical protein PF07 0110 - Plasmodium ... |
| **27** | Q38AF9 | Q38AF9_9TRYP | Hypothetical protein - Trypanosoma brucei |
| **28** | Q38EW0 | Q38EW0_9TRYP | Hypothetical protein - Trypanosoma brucei |
| **29** | Q38BI2 | Q38BI2_9TRYP | Hypothetical protein - Trypanosoma brucei |
| **30** | Q389J5 | Q389J5_9TRYP | Hypothetical protein - Trypanosoma brucei |
| **31** | Q38EV9 | Q38EV9_9TRYP | Hypothetical protein - Trypanosoma brucei |
| **32** | Q38DA6 | Q38DA6_9TRYP | Hypothetical protein - Trypanosoma brucei |
| **33** | Q57ZN0 | Q57ZN0_9TRYP | Hypothetical protein - Trypanosoma brucei |
| **34** | Q389J5 | Q389J5_9TRYP | Hypothetical protein - Trypanosoma brucei |
| **35** | Q388H5 | Q388H5_9TRYP | Hypothetical protein - Trypanosoma brucei |
| **36** | Q38BI2 | Q38BI2_9TRYP | Hypothetical protein - Trypanosoma brucei |
| **37** | Q57ZA0 | Q57ZA0_9TRYP | Hypothetical protein - Trypanosoma brucei |
| **38** | Tp_10241 |  |  |
| **39** | Tp_104814 | |  |
| **40** | Tp_106115 | |  |
| **41** | Tp_140452 | |  |
| **42** | Q8LL04 | Q8LL04_ARATH | CTD phosphatase-like 3 - Arabidopsis thalian... |
| **43** | Q9LJR5 | Q9LJR5_ARATH | Gb AAF34842.1 - Arabidopsis thaliana (Mouse-... |
| **44** | Q9LUP3 | Q9LUP3_ARATH | Gb AAD25584.1 - Arabidopsis thaliana (Mouse-... |
| **45** | Q9LJN7 | Q9LJN7_ARATH | Gb AAD25584.1 - Arabidopsis thaliana (Mouse-... |
| **46** | Q9LN24 | Q9LN24_ARATH | F14O10.8 protein - Arabidopsis thaliana (Mou... |
| **47** | Q9FHL1 | Q9FHL1_ARATH | Gb AAC78512.1 - Arabidopsis thaliana (Mouse-... |
| **48** | Q9FL74 | Q9FL74_ARATH | Gb AAF34842.1 - Arabidopsis thaliana (Mouse-... |
| **49** | Q00IB6 | Q00IB6_ARATH | Carboxyl-terminal phosphatase-like 4 - Arabi... |
| **50** | Q3ECX9 | Q3ECX9_ARATH | Protein At1g43610 (NLI interacting factor fa... |
| **51** | Q3ECX9 | Q3ECX9_ARATH | Protein At1g43610 (NLI interacting factor fa... |
| **52** | Q9ZVR2 | Q9ZVR2_ARATH | Hypothetical protein At2g02290 - Arabidopsis... |
| **53** | Q9SI33 | Q9SI33_ARATH | Hypothetical protein At2g04930 - Arabidopsis... |
| **54** | Q75JA4 | Q75JA4_DICDI | Similar to putative TFIIF-interacting compon... |
| **55** | Q9Y5B0 | CTDP1_HUMAN | RNA polymerase II subunit A C-terminal domain... |
| **56** | Q8IJR8 | Q8IJR8_PLAF7 | Hypothetical protein - Plasmodium falciparum... |
| **57** | Q8IDE5 | Q8IDE5_PLAF7 | Hypothetical protein MAL13P1.275 - Plasmodiu... |
| **58** | Tp_109700 | |  |
| **59** | Q55C70 | Q55C70_DICDI | Hypothetical protein - Dictyostelium discoid... |

Additional file 4.

Sequences of the NIF group retrieved from the genomic databases using the PF03031 Pfam profile, with their database annotation. The organisms from which the sequences originate are colour-coded as follows: red, *P. falciparum* (Alveolates); green*, A. thaliana* (Plants); blue, *H. Sapiens* (Opisthokonts); turquoise, *G. lamblia* (Excavates); purple, *T. brucei* (Discicristates); black, *T. pseudonana* (Heterokonts); and magenta, *D. discoideum* (Amoebozoa). See text for details.
