# Supplementary material for: The protein-phosphatome of the human malaria parasite Plasmodium falciparum
Source: BMC Genomics. 2008 Sep 15;9:412. doi: 10.1186/1471-2164-9-412 (PMC2559854; doi:10.1186/1471-2164-9-412)
Supplement: Additional file 5 — List of Rhodanese-conformant sequences. See legend within the file. [file 1471-2164-9-412-S5.doc]

| **1** | Q9LMU3 | ARATH | F2H15.8 protein - Arabidopsis thaliana (Mouse-ear cress) |
| --- | --- | --- | --- |
| **2** | Q56W89 | ARATH | Rhodanese like protein - Arabidopsis thaliana (Mouse-ear cress) |
| **3** | Q8RY72 | ARATH | Putative rhodanese family protein - Arabidopsis thaliana (Mouse-ear cress) |
| **4** | O48529 | ARATH | Rhodanese-like family protein - Arabidopsis thaliana (Mouse-ear cress) |
| **5** | Q501G8 | ARATH | At4g24750 - Arabidopsis thaliana (Mouse-ear cress) |
| **6** | Q94AC1 | ARATH | At1g09280/T12M41 - Arabidopsis thaliana (Mouse-ear cress) |
| **7** | Q5T7X0 | HUMAN | Chromosome 9 open reading frame 97 - Homo sapiens (Human) |
| **8** | tl0123 |  |  |
| **9** | tl0124 |  |  |
| **10** | tl0127 |  |  |
| **11** | tl0128 |  |  |
| **12** | tl0129 |  |  |
| **13** | tl0132 |  |  |
| **14** | tl0133 |  |  |
| **15** | tl0135 |  |  |
| **16** | tl0136 |  |  |
| **17** | tl0137 |  |  |
| **18** | Q16828 | HUMAN | Dual specificity protein phosphatase 7 - Homo sapiens(Human) |
| **19** | Q16829 | HUMAN | Dual specificity protein phosphatase 7 - Homo sapiens(Human) |
| **20** | Q5T603 | HUMAN | Dual specificity protein phosphatase 5 - Homo sapiens(Human) |
| **21** | Q86SS8 | HUMAN | Dual specificity phosphatase 8 - Homo sapiens (Human) |
| **22** | P28562 | HUMAN | Dual specificity protein phosphatase 1 - Homo sapiens(Human) |
| **23** | Q8IVT8 | HUMAN | DUSP16 protein - Homo sapiens (Human) |
| **24** | Q13115 | HUMAN | Dual specificity protein phosphatase 4 - Homo sapiens(Human) |
| **25** | Q05923 | HUMAN | Dual specificity protein phosphatase 2 - Homo sapiens(Human) |
| **26** | Q9Y6W6 | HUMAN | Dual specificity protein phosphatase 10 - Homo sapiens(Human) |
| **27** | Q99956 | HUMAN | Dual specificity protein phosphatase 9 |
| **28** | Q05DF5 | HUMAN | Ubiquitin carboxyl-terminal hydrolase 8 (USP8) - Homo sapiens (Human) |
| **29** | Q55GF0 | DICDI | Putative uncharacterized protein - Dictyostelium discoideum (Slime mold) |
| **30** | Q86KQ2 | DICDI | Putative uncharacterized protein - Dictyostelium discoideum (Slime mold) |
| **31** | Q8NFU3 | HUMAN | Putative thiosulfate sulfurtransferase KAT - Homo sapiens(Human) |
| **32** | Q382H5 | 9TRYP | Putative uncharacterized protein - Trypanosoma brucei |
| **33** | Q57W37 | 9TRYP | Putative uncharacterized protein - Trypanosoma brucei |
| **34** | Q382H6 | 9TRYP | Putative uncharacterized protein - Trypanosoma brucei |
| **35** | Q586G5 | 9TRYP | Putative uncharacterized protein - Trypanosoma brucei |
| **36** | tl0125 |  |  |
| **37** | tl0131 |  |  |
| **38** | Q9M4F7 | ARATH | 3-mercaptopyruvate sulfurtransferase precursor - Arabidopsis thaliana (Mouse-ear cress) |
| **39** | Q9SCY8 | ARATH | 3-mercaptopyruvate sulfurtransferase precursor - Arabidopsis thaliana (Mouse-ear cress) |
| **40** | Q54E40 | DICDI | Putative uncharacterized protein - Dictyostelium discoideum (Slime mold) |
| **41** | A8BJL6 | GIALA | Thiosulfate sulfurtransferase - Giardia lamblia ATCC 50803 |
| **42** | Q16762 | HUMAN | Thiosulfate sulfurtransferase - Homo sapiens (Human) |
| **43** | P25325 | HUMAN | 3-mercaptopyruvate sulfurtransferase - Homo sapiens(Human) |
| **44** | Q582K7 | 9TRYP | Mercaptopyruvate sulfurtransferase, putative - Trypanosoma brucei |
| **45** | tl0122 |  |  |
| **46** | tl0130 |  |  |
| **47** | Q8LEW3 | ARATH | Senescence-associated protein sen1 - Arabidopsis thaliana (Mouse-ear cress) |
| **48** | Q8RUD6 | ARATH | Senescence-associated protein - Arabidopsis thaliana (Mouse-ear cress) |
| **49** | Q9FKW8 | ARATH | Senescence-associated protein sen1-like protein - Arabidopsis thaliana (Mouse-ear cress) |
| **50** | Q7XJR6 | ARATH | Putative senescence-associated rhodanese protein - Arabidopsis thaliana (Mouse-ear cress) |
| **51** | Q39129 | ARATH | Thiosulfate sulfurtransferase(STR16) - Arabidopsis thaliana (Mouse-ear cress) |
| **52** | tl0134 |  |  |
| **53** | tl0138 |  |  |
| **54** | Q86M49 | DICDI | Tyrosine phosphatase CDC25 - Dictyostelium discoideum (Slime mold) |
| **55** | P30305 | HUMAN | M-phase inducer phosphatase 2 - Homo sapiens (Human) |
| **56** | P30307 | HUMAN | M-phase inducer phosphatase 3 - Homo sapiens (Human) |
| **57** | P30304 | HUMAN | M-phase inducer phosphatase 1 - Homo sapiens (Human) |
| **58** | Q8IER0 | PLAF7 | Putative uncharacterized protein PF13_0027 - Plasmodium falciparum (isolate 3D7) |
| **59** | Q38EP7 | 9TRYP | Putative uncharacterized protein - Trypanosoma brucei |
| **60** | Q585K9 | 9TRYP | Putative uncharacterized protein - Trypanosoma brucei |
| **61** | Q389B6 | 9TRYP | Putative uncharacterized protein - Trypanosoma brucei |
| **62** | Q581R0 | 9TRYP | Putative uncharacterized protein - Trypanosoma brucei |
| **63** | Q8GY31 | ARATH | Dual specificity phosphatase Cdc25 - Arabidopsis thaliana (Mouse-ear cress) |
| **64** | Q9T092 | ARATH | Putative uncharacterized protein AT4g27700 - Arabidopsis thaliana (Mouse-ear cress) |
| **65** | Q8LA89 | ARATH | Putative uncharacterized protein - Arabidopsis thaliana (Mouse-ear cress) |
| **66** | Q0WWP1 | ARATH | Putative uncharacterized protein At4g01050 - Arabidopsis thaliana (Mouse-ear cress) |
| **67** | Q9FN48 | ARATH | Emb CAB75797.1 - Arabidopsis thaliana (Mouse-ear cress) |
| **68** | Q556Y8 | DICDI | Putative uncharacterized protein - Dictyostelium discoideum (Slime mold) |
| **69** | Q54Y84 | DICDI | Putative uncharacterized protein - Dictyostelium discoideum (Slime mold) |
| **70** | Q54NS5 | DICDI | Putative uncharacterized protein - Dictyostelium discoideum (Slime mold) |
| **71** | A8B3D6 | GIALA | Dual specificity phosphatase Cdc25, putative - Giardiam lamblia ATCC 50803 |
| **72** | A8B373 | GIALA | Putative uncharacterized protein - Giardia lamblia ATCC 50803 |
| **73** | A8B236 | GIALA | Putative uncharacterized protein - Giardia lamblia ATCC 50803 |
| **74** | A8BED4 | GIALA | Putative uncharacterized protein - Giardiam lamblia ATCC 50803 |
| **75** | Q8TEA7 | HUMAN | TBC domain-containing protein kinase-like protein - Homo sapiens (Human) |
| **76** | Q9Y6J8 | HUMAN | Serine/threonine/tyrosine-interacting-like protein 1 (Dual specificity protein phosphatase 24) (Map kinase phosphatase-like protein MK-STYX) |
| **77** | Q9BYV8 | HUMAN | Centrosomal protein of 41 kDa - Homo sapiens (Human) |
| **78** | Q9NUY8 | HUMAN | TBC1 domain family member 23 |
| **79** | Q8I5Y1 | PLAF7 | (isolate 3D7) Putative uncharacterized protein PFL0320w - Plasmodium falciparum (contains 2 Rhodanese domains) |
| **80** | tl0126 |  |  |

Additional file 5.

Sequences of the Rhodanese group retrieved from the genomic databases using the PF00581 Pfam profile, with their database annotation. The organisms from which the sequences originate are colour-coded as follows: red, *P. falciparum* (Alveolates); green*, A. thaliana* (Plants); blue, *H. Sapiens* (Opisthokonts); turquoise, *G. lamblia* (Excavates); purple, *T. brucei* (Discicristates); black, *T. pseudonana* (Heterokonts); and magenta, *D. discoideum* (Amoebozoa). See text for details.
