# Supplementary figures and images for: The protein-phosphatome of the human malaria parasite Plasmodium falciparum
Source: BMC Genomics. 2008 Sep 15;9:412. doi: 10.1186/1471-2164-9-412 (PMC2559854; doi:10.1186/1471-2164-9-412)

## A

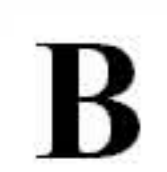

Supplement: Additional file 7 — High resolution file of Fig. 3. See legend to Fig. 3. [file 1471-2164-9-412-S7.pdf]

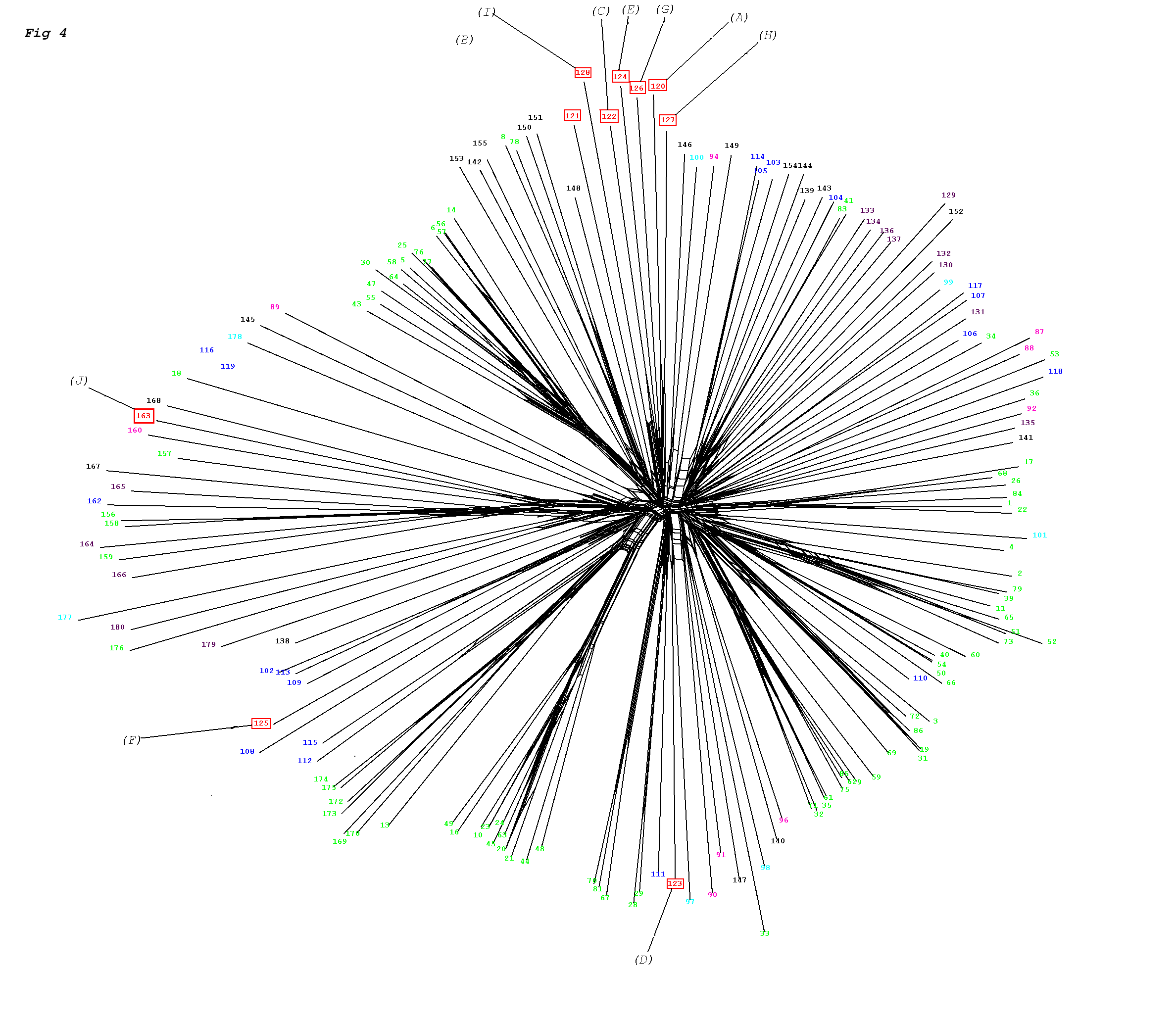

Supplement: Additional file 8 — High resolution file of Fig. 4. See legend to Fig. 4. [file 1471-2164-9-412-S8.png]

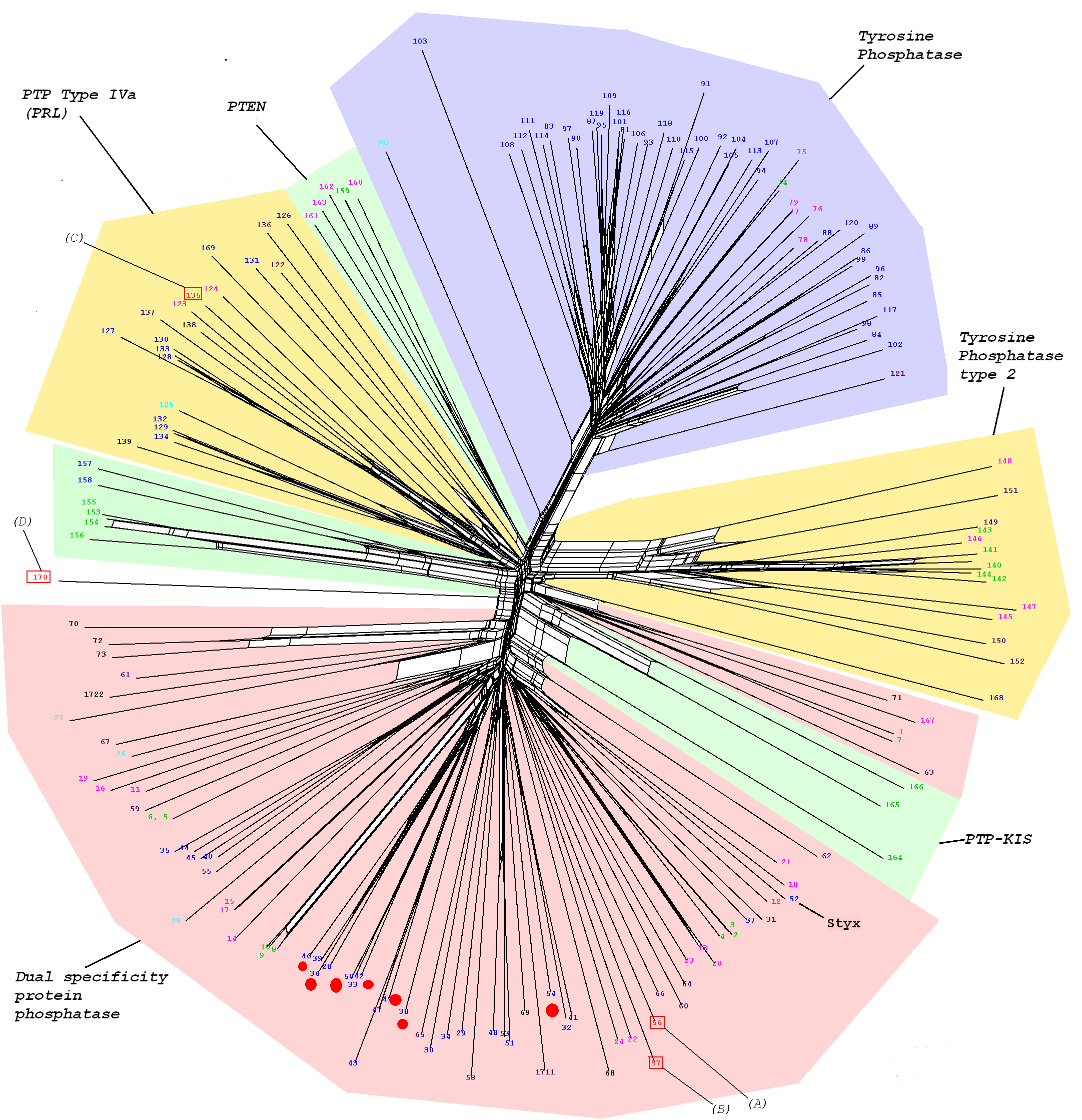

Supplement: Additional file 9 — High resolution file of Fig. 5. See legend to Fig. 5 [file 1471-2164-9-412-S9.png]

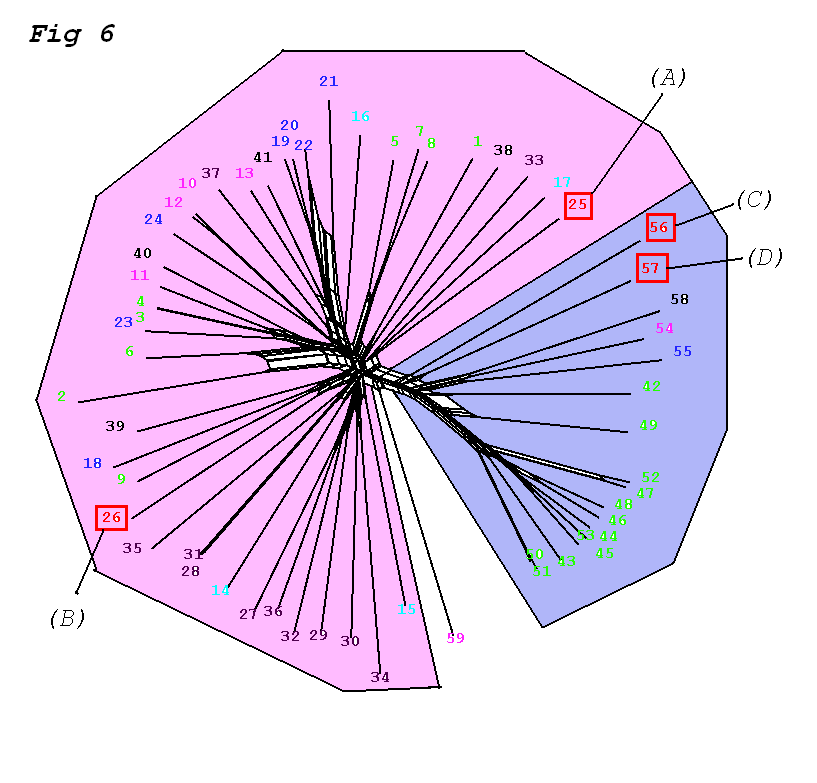

Supplement: Additional file 10 — High resolution file of Fig. 6. See legend to Fig. 6. [file 1471-2164-9-412-S10.png]

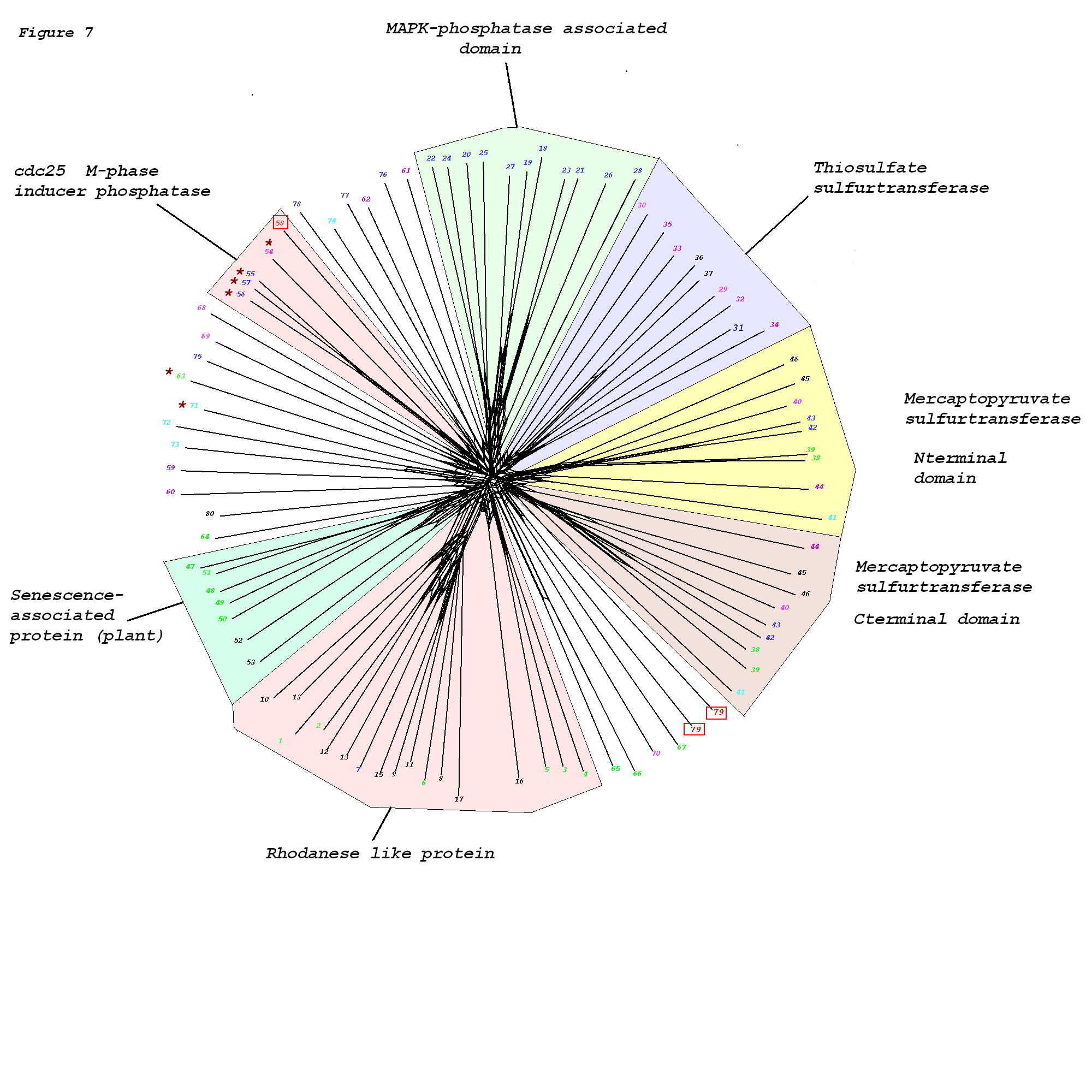

Supplement: Additional file 11 — High resolution file of Fig. 7. See legend to Fig. 7. [file 1471-2164-9-412-S11.png]
