# Supplementary material for: The protein-phosphatome of the human malaria parasite Plasmodium falciparum
Source: BMC Genomics. 2008 Sep 15;9:412. doi: 10.1186/1471-2164-9-412 (PMC2559854; doi:10.1186/1471-2164-9-412)
Supplement: Additional file 6 — Alignment of Rhodanese-containing domains. See legend within the file. [file 1471-2164-9-412-S6.doc]

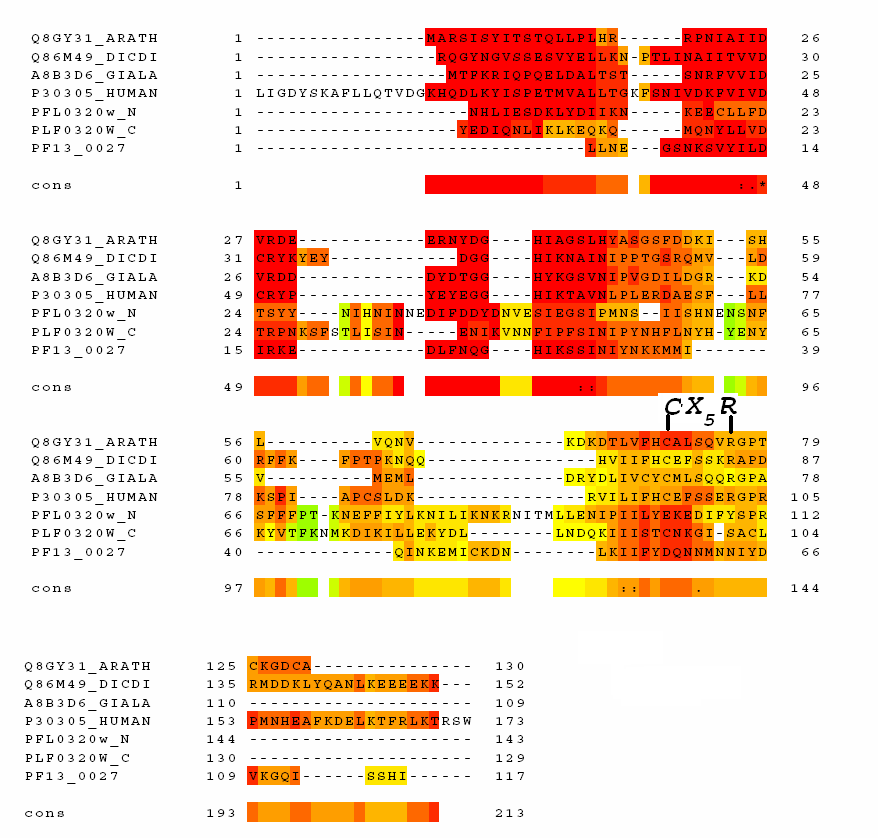


Additional file 6.

Multiple Sequence Alignment (T-coffee) of catalytic domains of Cdc25 (conformant to the Pfam PF00581 [Rhodanese] profile) from *H. sapiens*, *A. thaliana, D. discoideum* and *G. lamblia* along with the PF00581-conformant sequences from *P. falciparum*. The **CX_5_R** motif of the catalytic site is indicated. Note that no *P. falciparum* sequence possesses this motif.
